# Combining remote sensing with local knowledge is vital for understanding forest change in West Africa

**DOI:** 10.1038/s41598-025-23133-5

**Published:** 2025-10-30

**Authors:** Chima Jude Iheaturu, Felicia Olufunmilayo Akinyemi, Vladimir Ruslan Wingate, Paule Pamela Tabi Eckebil, Chinwe Ifejika Speranza

**Affiliations:** 1 Land Systems and Sustainable Land Management, Institute of Geography, University of Bern, Hallerstrasse 12, 3012 Bern, Switzerland; 2 Geomatics, Department of Environmental and Life Sciences, Karlstad University, Universitetsgatan 2, 651 88 Karlstad, Sweden

**Keywords:** Ecology, Environmental sciences, Environmental social sciences

## Abstract

Understanding tropical forest change requires integrating satellite observations with insights from forest-dependent communities. In West Africa, where deforestation and degradation unfold within complex social-ecological systems, conventional monitoring often overlooks community insights. We combined Landsat-derived forest cover data (2000–2022) with household surveys from 2,621 respondents across nine forest patches, applying a convergence matrix to compare satellite trajectories with local knowledge of forest change. Two themes were analyzed: forest cover loss and forest regrowth. Sites were classified as full convergence, partial convergence, or dissonance based on directional agreement and the proportion of community responses. Full convergence occurred in four sites where > 65% of respondents reported forest loss consistent with satellite-detected declines (− 2.8% to − 13.9% cover). Partial convergence characterized the two sites with mixed local responses and modest net satellite-image changes (< 5%). Dissonance emerged in three sites where satellite-detected stability (< 2% net change, p ≥ 0.10) contrasted with > 65% of respondents reporting degradation. Across all sites, satellite-detected regrowth was minimal, though some communities described localized recovery. These findings show that convergence is strongest for deforestation, while divergences are concentrated around degradation, underscoring the diagnostic value of local knowledge and the limitations of medium-resolution imagery. Embedding such knowledge in monitoring frameworks is therefore both an ethical imperative and a strategic necessity for adaptive forest governance.

**Supplementary Information:**

The online version contains supplementary material available at 10.1038/s41598-025-23133-5.

## Introduction

Tropical forests buffer global climate change, harbor over half of terrestrial biodiversity and sustain the livelihoods of hundreds of millions of people^1^. Yet they are disappearing faster than any other major biome^2^. In West Africa, where population densities are high and agrarian economies dominate, annual tree‑cover loss exceeded 1.9 million hectares between 2015 and 2020, ranking among the steepest rates worldwide^3^. Such losses jeopardize regional targets for climate mitigation, biodiversity conservation and human well‑being, but their full extent and underlying mechanisms remain poorly resolved.

Satellite remote sensing has transformed our ability to monitor forest dynamics at continental scales^4^. Time‑series analyses of Landsat imagery now routinely quantify where canopies are cleared, fragmented or undergoing regrowth^5–7^. These data underpin global carbon accounting frameworks and early‑warning platforms such as Global Forest Watch (GFW). However, medium‑resolution sensors (10–30 m) struggle to detect “cryptic” degradation—canopy thinning, selective logging and understory clearing—that often precedes outright deforestation^8–10^. More fundamentally, spectral trajectories reveal little about the social, political and cultural drivers that shape forest trajectories. Without such context, technocratic assessments risk misdiagnosing change and prescribing maladaptive or unjust interventions^11^.

A growing body of scholarship therefore argues for knowledge pluralism: the systematic integration of spatially explicit observations with the situated expertise of forest‑dependent communities^12,13^. Ethnographic and participatory studies across the tropics show that local actors observe ecological change through lenses of tenure security, livelihood risk and historical memory, often detecting early degradation long before it becomes spectrally visible^14–16^. Yet empirical demonstrations of how satellite evidence and local knowledge converge, or diverge, remain scarce, particularly in Africa, where multi-country comparisons are limited and methodological templates for integration are lacking.

We bridge this gap by combining two decades of satellite-derived data with 2,621 household surveys across nine forest patches in Togo, Benin, Nigeria and Cameroon (See Supplementary Table 1). The study sites represent diverse governance types from purely community-managed forests to mixed community-family tenure systems, distributed across a bioclimatic transition from subhumid warm ecosystems in Togo and Benin to humid warm forests in Nigeria and Cameroon. The selection of these nine sites was guided by the archetypes of remnant West African forest patches identified by Wingate et al.^17^, which group patches according to recurrent biophysical and social-ecological characteristics. This ensures representativeness of the main regional forest change dynamics. In addition, practical considerations such as accessibility, availability of historical data, and feasibility of community engagement also shaped the final sample.

We deploy a convergence matrix that pairs satellite‑derived trajectories of forest cover (2000–2022) with local knowledge of change over matched recall periods. Our analysis addresses three questions: (1) Where do satellite observations and local knowledge corroborate one another, thereby boosting confidence in diagnoses of deforestation, stability, or regrowth? (2) Where do they diverge, and what ecological processes (e.g., canopy thinning, natural regeneration) or socio‑political dynamics (e.g., weak governance, effective law enforcement) explain the discrepancy?; and (3) How can hotspots of convergence or divergence inform more equitable monitoring, reporting and verification (MRV) systems in Reducing Emissions from Deforestation and forest Degradation (REDD +) and guide context‑sensitive conservation strategies?

Here, we show that capturing tropical forest change is not only a technical challenge of better sensors but an epistemological endeavor that hinges different data and perspectives. Satellites in the sky and stories on the ground provide complementary, and occasionally competing, views of forest change reality. Only by weaving them together can we capture the multi‑scalar and multi-dimensional dynamics that shape the future of West Africa’s forests and craft interventions that are both scientifically robust and socially just.

## Results

### Spatiotemporal patterns of forest cover change (2000–2022)

**Fig. 1:**
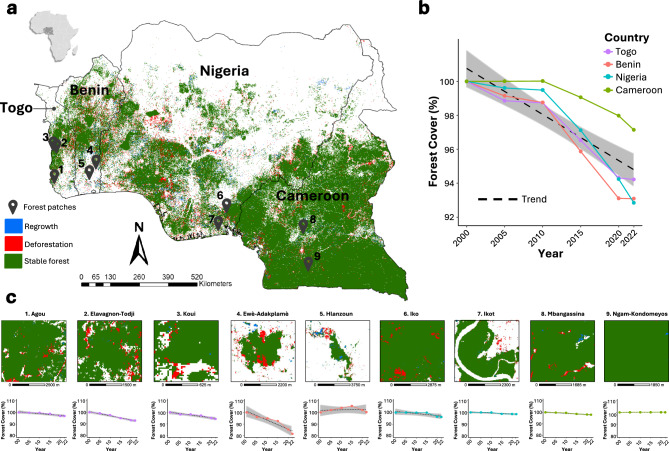
Spatiotemporal patterns of forest cover change across the study area (2000–2022). (**a**) Regional map derived from Landsat imagery obtained from Google Earth Engine (GEE, https://earthengine.google.com/) classifies each 30 m pixel as net deforestation (red), net regrowth (blue) or stable forest (green). (**b**) National trajectories for Togo, Benin, Nigeria and Cameroon show annual forest area, each normalized to its 2000 extent (set to 100%). (**c**) Forest cover trajectories for nine selected forest patches—Agou, Elavagnon-Todji, Koui (Togo); Ewè-Adakplamè, Hlanzoun, also known as Lokoli (Benin); Iko, Ikot (Nigeria); and Mbangassina, Ngam-Kondomeyos (Cameroon)—with area normalized to year 2000 extent (set to 100%). Forest cover trends are expressed as annual percentages relative to baseline (year 2000), and fitted with linear models (dashed lines) to reveal overall direction and magnitude of change. Maps created by the authors using ArcGIS Pro v 3.5.2 (https://www.esri.com/en-us/arcgis/products/arcgis-pro/overview). See Supplementary Table 2 for patch-specific forest extent.

Our Landsat-derived analysis reveals that forest cover across the nine West African sites declined markedly between 2000 and 2022, with region-wide net forest loss reaching 13.3%. However, the pace, direction, and character of change varied widely across countries and forest patches (Fig. 1).

At the national scale (Fig. 1b), Nigeria recorded the greatest absolute forest loss—approximately 1.55 million hectares—followed by Cameroon (1.02 Mha), Benin (0.34 Mha), and Togo (0.13 Mha), corresponding to average annual deforestation rates of 0.33%, 0.13%, 0.31%, and 0.26%, respectively. These aggregate trajectories mask significant heterogeneity at the patch level (Fig. 1c). Among the nine focal sites, Ewè-Adakplamè (Benin) exhibited the steepest decline, with forest area shrinking by 18.4% (619 ha to 505 ha). Relatively moderate declines were also observed in Elavagnon-Todji and Agou (Togo), at 6.8% and 3.2%, respectively.

In contrast, Hlanzoun (Benin) displayed relative stability, with minor fluctuations around a near-constant baseline (547 ha in 2000 to 549 ha in 2022). Similar persistence was observed in Cameroon’s Mbangassina and Ngam-Kondomeyos, where forest area remained effectively unchanged over two decades—suggesting effective local stewardship, low disturbance rates, or potential under-detection of small-scale degradation. Notably, Ikot (Nigeria) also maintained a relatively stable canopy cover (< 1% net loss), though adjacent land use shifted.

**Fig. 2:**
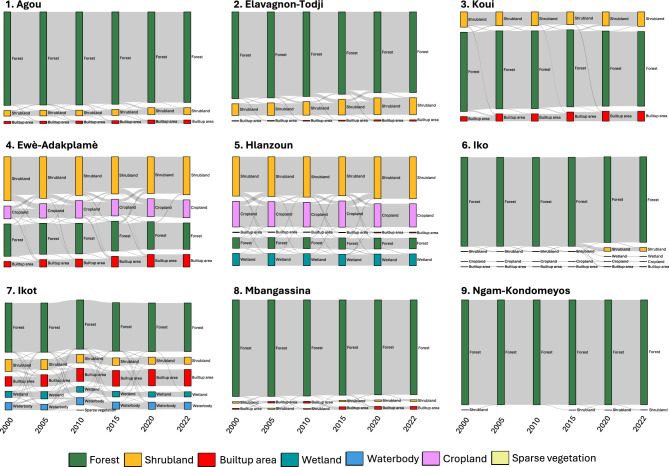
Land use and land cover (LULC) transitions across nine forest patches in the study area (2000–2022). Sankey diagrams illustrate temporal dynamics of LULC across the nine forest patches studied: **1** Agou, **2** Elavagnon-Todji, **3** Koui (Togo); **4** Ewè-Adakplamè, **5** Hlanzoun (Benin); **6** Iko, **7** Ikot (Nigeria); and **8** Mbangassina, **9** Ngam-Kondomeyos (Cameroon). Transitions are shown across five time intervals—2000–2005, 2005–2010, 2010–2015, 2015–2020, and 2020–2022. Flow widths are proportional to the area of transition (in hectares) between land cover classes (See Supplementary Table 3 for details). Color coding denotes LULC categories, including forest, shrubland, cropland, built-up areas, wetland, waterbody, and sparse vegetation. Together, the diagrams reveal patch-specific pathways of forest loss, degradation, and regrowth.

Temporal land-use trajectories highlight the processes underpinning these trends (Fig. 2). Forest-to-shrubland transitions were common in sites such as Agou and Iko, indicating progressive degradation rather than abrupt clearance. For example, Agou lost 40.77 ha to shrubland between 2015–2020 alone, while Iko registered 128.7 ha of forest degradation post-2015. Meanwhile, smaller regrowth gains (e.g., 9.81 ha of shrubland reverting to forest in Agou) suggest fallow cycling or ecological recovery under shifting cultivation.

Ewè-Adakplamè presented complex, cyclical transitions among shrubland, cropland, and built-up areas. From 2000–2015, over 100 ha of shrubland were converted to cropland, followed by a surge in urban expansion. In Hlanzoun (Lokoli), dynamic forest–wetland exchanges were observed, likely driven by hydrological variability and small-scale cropland encroachment. For instance, 21.96 ha transitioned from wetland to forest (2010–2015), while 6.66 ha of wetland were lost to cropland.

Patch-level variation was equally evident in Cameroon. Mbangassina experienced minor forest losses (15.6 ha), largely attributable to shrub encroachment and modest expansion of built-up areas. Ngam-Kondomeyos remained the most stable site, with forest cover persistently near 1,284 ha and minimal transition activity—indicating either successful governance or low external pressure.

Collectively, these results reveal three forest-change archetypes: (1) consistent, high-magnitude decline (e.g., Ewè-Adakplamè); (2) relative canopy stability with dynamic edge processes (e.g., Hlanzoun, Ikot); and (3) minimal change and potential conservation success (e.g., Ngam-Kondomeyos).

### Demographic characteristics of forest-adjacent communities

**Fig. 3:**
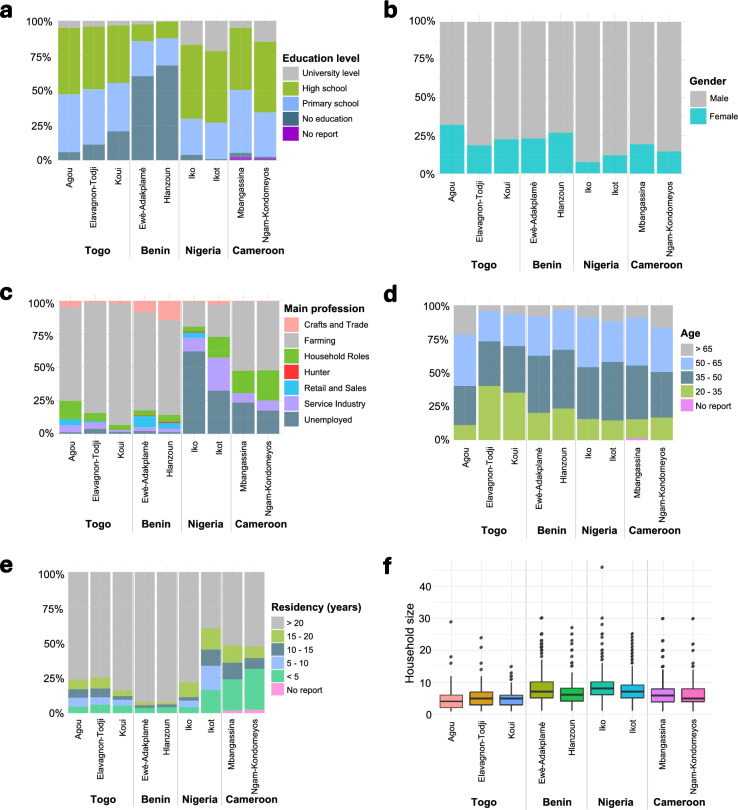
Demographic characteristics of household survey respondents across forest-adjacent communities in Togo, Benin, Nigeria, and Cameroon. (**a**) Highest education level attained (categorized as no formal education, primary, secondary/high school, tertiary), (**b**) Gender distribution (percentage of male and female respondents), (**c**) Main occupation/profession of respondents (categorized as crafts and trades, farming, household roles, hunters, unemployed, or others), (**d**) Age distribution (categorized into age brackets: 20–35, 35–50, 50–65, and > 65 years), (**e**) Residency duration in community (categorized as < 5, 5–10, 10–15, 15–20, and > 20 years), and (**f**) Household size (number of individuals per household). Data were collected from a total of 2,621 households surveyed between October 2022 and March 2023 across nine forest patches: Agou (n = 351), Elavagnon-Todji (n = 306), and Koui (n = 150) in Togo; Ewè-Adakplamè (n = 307) and Hlanzoun (n = 312) in Benin; Iko (n = 344) and Ikot (n = 377) in Nigeria; and Mbangassina (n = 302) and Ngam-Kondomeyos (n = 172) in Cameroon.

The surveyed communities adjacent to the nine forest patches exhibited significant demographic variability regarding household sizes, residency durations, gender distributions, age profiles, education, and primary occupations (Fig. 3).

Gender composition among respondents was predominantly male across all sites, though notable differences emerged between communities (Fig. 3b). Male participation was highest in Nigeria’s Iko forest (93%), whereas Togo’s Agou forest exhibited a relatively balanced gender ratio (68% male; 32% female). Other sites, such as Cameroon’s Ngam-Kondomeyos forest, similarly showed strong male dominance (85% male), reflecting widespread patterns of gendered household representation.

The age distribution highlighted a predominantly middle-aged demography across all study sites (Fig. 3d). In Benin’s Hlanzoun and Ewè-Adakplamè forests, approximately 73% and 71% of respondents, respectively, were aged between 35 and 65 years. Togo’s forest sites displayed similar patterns, with Agou (67%), Koui (58%), and Elavagnon-Todji (55%) having a majority within this middle-aged bracket. In Nigeria, the Ikot forest had a slightly younger profile (44% aged 35–50 years), while Iko displayed a more even distribution across age groups, including notable proportions in both the 35–50 years (38%) and 50–65 years (37%) brackets. Cameroon’s Mbangassina and Ngam-Kondomeyos forests were comparable, with most respondents aged between 35 and 65 years.

Educational attainment varied considerably between communities (Fig. 3a). Respondents in Togo’s Agou (48%), Nigeria’s Iko (53%), and Cameroon’s Ngam-Kondomeyos forests (51%) predominantly reported completion of high school (secondary education). In contrast, high proportions of respondents with no formal education were recorded in Benin’s Ewè-Adakplamè (61%) and Hlanzoun forests (68%), highlighting significant disparities in educational access and attainment.

Household sizes exhibited notable variability across the study region (Fig. 3f). The largest average household sizes were reported in Nigeria’s Iko forest and Benin’s Ewè-Adakplamè forest (mean = 8 individuals). Smaller household sizes characterized Togo’s forest sites: Agou (mean = 4), Koui (mean = 5), and Elavagnon-Todji (mean = 5).

Residency durations revealed distinct settlement stability patterns (Fig. 3e). Long-term residency (over 20 years) was predominant (> 90%) in Benin’s Ewè-Adakplamè and Hlanzoun forests, indicative of stable community dynamics. Conversely, Nigeria’s Ikot forest exhibited more transient settlement patterns, with only 40% of respondents residing for more than 20 years, and 33% for less than 10 years. Cameroon’s Ngam-Kondomeyos forest similarly showed high mobility, with 29% of respondents reporting residence durations of less than five years, reflecting a recently increasing settlement population.

### Local knowledge of forest cover change

**Fig. 4:**
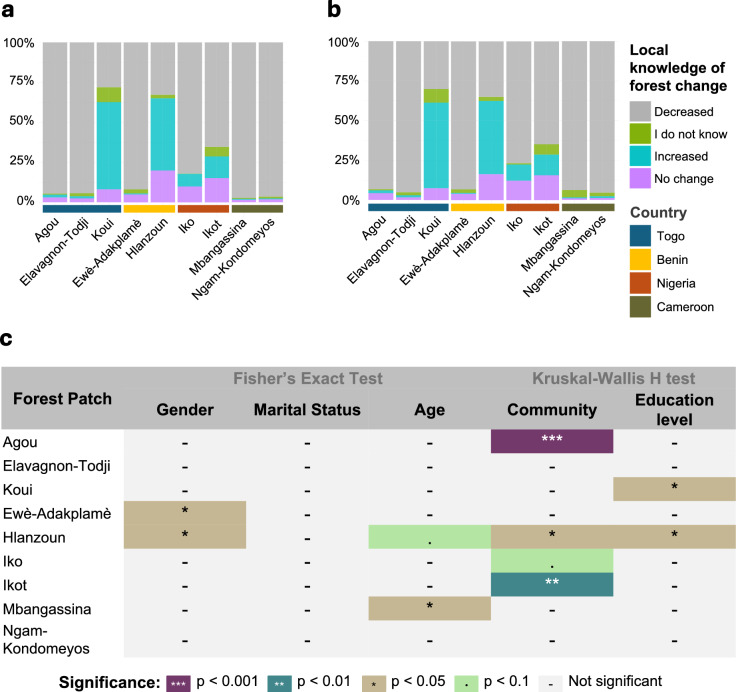
Local knowledge and socio-demographic variation in reported forest change across the nine forest-adjacent landscapes. (**a**) Local knowledge of forest cover change over the past five years (2017–2022), categorized as Decreased, I do not know, Increased, or No change, aggregated at the forest patch level. (**b**) Local knowledge of forest cover change over the past ten years (2012–2022), similarly categorized. (**c**) Statistical significance of variation in local knowledge by gender, marital status, age, community, and education level, based on Fisher’s Exact Test and Kruskal–Wallis H test results (See Supplementary Table 4 for details).

Across all nine forest patches studied, most respondents reported local knowledge indicating a decline in forest cover over both five- and ten-year periods, although spatial patterns of reported change and the socio-demographic factors influencing this knowledge varied by site (Fig. 4).

In the Agou forest (Togo), 95% of respondents reported forest decline over the past five years, and 93% over the past ten years. Similarly high proportions were observed in Elavagnon-Todji, where 94% and 95% of respondents reported declines over five- and ten-year periods, respectively. Statistical analysis revealed significant variation in local knowledge across communities in Agou (p < 0.001), but not in Elavagnon-Todji (p = 0.368), where responses were more consistent.

Local knowledge in Koui (Togo) diverged from this broader trend: 55% of respondents reported an increase in forest cover over five years, and 54% over ten years, while 28–30% reported declines. Education level was significantly associated with local knowledge (p = 0.034), with respondents having primary or secondary education more likely to report increases in forest cover.

In Benin, local knowledge around Hlanzoun were mixed. Over five years, 45% of respondents reported increased forest cover, and 33% reported decline. Over ten years, 46% observed an increase and 35% a decline. Gender was a significant factor influencing responses (p = 0.047), and significant differences were also observed across communities (p = 0.046) and education levels (p = 0.014). In contrast, in Ewè-Adakplamè, local knowledge of forest decline were near unanimous: 92% and 93% of respondents reported losses over five and ten years, respectively. Gender was a significant factor (p = 0.031), while no differences were found across communities (p = 0.783) or education levels (p = 0.795).

In Nigeria, respondents in Iko forest overwhelmingly reported forest decline (82% over five years; 77% over ten years), while in Ikot, 65% reported losses across both timeframes. Although demographic variables such as gender, age, and marital status were not statistically significant (p > 0.05), community-level variation in Ikot was notable (p = 0.0099), suggesting differentiated local experiences.

In Cameroon, respondents around Ngam-Kondomeyos reported the highest levels of local knowledge of forest loss, with 97% and 95% indicating decline over five and ten years, respectively. Mbangassina showed similarly strong reports of decline (97% over five years; 94% over ten years). While most socio-demographic factors did not significantly influence responses in either site (p > 0.05), local knowledge in Mbangassina varied significantly by age group (p = 0.022), indicating possible generational differences in environmental memory or forest interaction.

These patterns highlight both the widespread local knowledge of deforestation and the influence of local socio-demographic context in shaping how forest change is understood by communities.

### Community-identified drivers of forest change

**Fig. 5:**
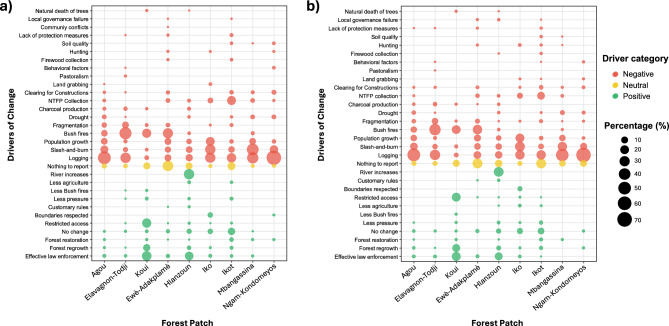
Community-Identified drivers of forest change across nine forest patches in the study area. (**a**) Drivers reported over a five-year recall period (2017–2022). (**b**) Drivers reported over a ten-year recall period (2012–2022). Each point represents a specific driver of forest change as identified by community members, with its size indicating the percentage of respondents citing that driver in each forest patch. Drivers are categorized as positive (green; associated with local knowledge of forest increase or stability, such as regrowth or effective law enforcement), neutral (yellow; indicating uncertainty such as “nothing to report”), or negative (red; associated with local knowledge of forest loss, such as logging, slash-and-burn agriculture, or population growth pressure).

Local knowledge revealed a complex array of socio-economic, environmental, and institutional factors driving forest change across the nine forest patches. While drivers varied across sites and recall periods (five years and ten years), negative drivers—particularly logging, slash-and-burn agriculture, bush fires, and population growth pressure—dominated local accounts. Fewer respondents identified positive or stabilizing influences such as forest regrowth, effective law enforcement, or access restrictions (Fig. 5).

In Togo, logging emerged as the primary identified driver in both Agou and Elavagnon-Todji, cited by over half of respondents in Agou (50% over five years, 53% over ten years) and nearly one-third in Elavagnon-Todji (30% and 32%). In Elavagnon-Todji, bush fires were cited even more frequently than logging (45% and 42%), while slash-and-burn agriculture and fragmentation were also mentioned. In both sites, mentions of forest restoration or regrowth were minimal. By contrast, respondents in Koui described a different dynamic: effective law enforcement (25% and 22%) and restricted access (23% and 21%) were reported as the most influential factors, alongside bush fires (21% and 20%) and forest regrowth (11% and 15%). These responses suggest that forest governance interventions may have supported local knowledge of stabilization or recovery.

In Benin, communities offered more varied explanations. In Ewè-Adakplamè, bush fires (28% over five years; 25% over ten) and logging (10% and 14%) were frequently cited, followed by population growth and slash-and-burn agriculture. However, a substantial share of respondents (31% and 27%) reported “nothing to report,” reflecting either uncertainty, disengagement, limited visibility of change, or reluctance to attribute blame. Notably, one respondent (0.3% during the five-year recall period) identified community conflict as a contributing factor, highlighting the potential—albeit rare—role of local social tensions in influencing forest outcomes. Hlanzoun presented a more mixed narrative: river expansion (30% and 27%) and effective law enforcement (19% and 15%) were among the most cited drivers, but logging (12–13%) and slash-and-burn agriculture (9%) remained relevant. Notably, respondents also cited forest regrowth, firewood collection, and customary rules, suggesting diverse experiences with forest dynamics.

In Nigeria, respondents in Iko predominantly attributed forest loss to slash-and-burn agriculture (30% and 28%), logging (29% and 24%), and population growth (20% and 21%). A minority mentioned boundaries being respected or forest regrowth, while a few indicated “nothing to report”. In Ikot, the narrative was slightly different: logging (23% and 25%) and non-timber forest products (NTFP) collection (21% and 14%) were identified as the dominant drivers, with smaller proportions citing slash-and-burn agriculture, clearing for construction, and forest restoration. The high proportion of neutral responses (15% and 27% indicating “nothing to report”) may point to a lower intensity of visible land-use changes or more spatially diffuse impacts.

In Cameroon, logging and slash-and-burn agriculture were identified as the primary drivers of forest change in both Mbangassina and Ngam-Kondomeyos. In Mbangassina, 41% and 24% of respondents cited slash-and-burn agriculture as the leading driver over the five- and ten-year periods, respectively, while 37% and 51% cited logging. Population growth, drought, and NTFP collection were also mentioned. In Ngam-Kondomeyos, the dominance of logging was particularly striking, with 68% (five-year) and 72% (ten-year) of respondents citing it as the principal cause of change. Other drivers such as slash-and-burn agriculture, clearing for construction, and drought were mentioned by smaller proportions, while neutral or positive drivers (e.g., forest regrowth, behavioral change) were cited rarely.

Overall, negative drivers such as logging, slash-and-burn agriculture and population growth were the most widely cited across sites and recall periods. However, respondents also acknowledged positive or stabilizing forces in select locations, particularly Koui and Hlanzoun, where enforcement, access restriction or ecological processes were linked to forest stability or recovery. Meanwhile, 'nothing to report’ responses, classified as neutral, varied by site and may reflect limited engagement, local disconnection from forest governance, the invisibility of gradual changes or reluctance to attribute blame.

### Convergence and divergence analysis

**Fig. 6:**
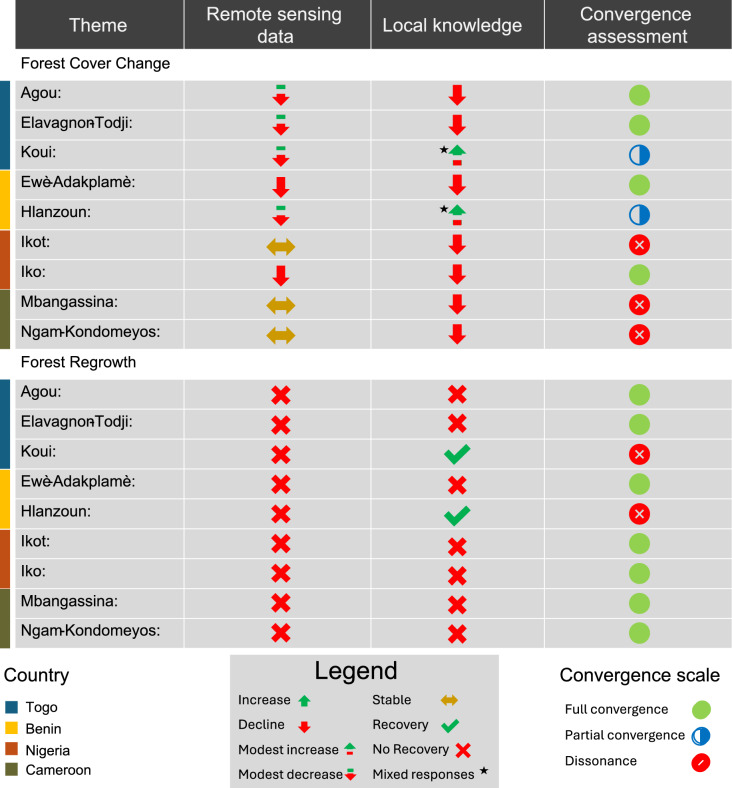
Convergence matrix comparing remote sensing data and local knowledge of forest change across nine forest patches in Western Africa. This matrix synthesizes thematic evidence from two sources—satellite-derived forest cover trends and community-reported observations—organized under two key themes: Forest Cover Change and Forest Regrowth. For each forest patch, insights from remote sensing and local knowledge are presented side by side. The final column, "Convergence assessment," classifies the degree of agreement between the two data sources as full convergence, partial convergence, or dissonance. Sites with mixed local responses are marked with an asterisk. Classification thresholds are defined as follows: more than 65 percent agreement indicates convergence or dissonance (depending on directional match or mismatch); 35 to 65 percent agreement indicates partial convergence; less than 35 percent agreement indicates weak or no convergence.

We compared forest cover dynamics derived from remote sensing with community-reported local knowledge of forest change across the nine forest patches examined. This comparison focused on the most recent decade (2010–2022) to match the ten-year recall period (2012–2022) used in the community surveys. Using a convergence matrix, we classified sites into three categories: full convergence, partial convergence and dissonance, based on directional agreement and the proportion of community responses (Fig. 6).

**Full convergence** was observed in sites where both remote sensing and local knowledge indicated the same direction of forest change, and more than 65 percent of respondents supported that direction. In Ewè-Adakplamè (Benin), Landsat data showed a steady decline in forest cover from 586.9 ha in 2010 to 505.4 ha in 2022, consistent with 93% of respondents who reported deforestation, primarily due to bush fires and logging. A similar pattern was found in Iko (Nigeria), where forest cover dropped from 3554.2 ha to 3423.5 ha after 2015, matching widespread reports of intensified slash-and-burn agriculture and logging. Elavagnon-Todji (Togo) also met the criteria for full convergence: satellite-derived forest loss (680.8 ha to 648.4 ha) corresponded with 95% of respondents reporting decline, attributing it to fire and fragmentation. Likewise, in Agou (Togo), modest forest loss (2346.0 ha to 2280.9 ha) was consistent with 93% of respondents who described major degradation linked to canopy thinning and edge encroachment. These cases demonstrate directional and majority agreement between data sources.

**Partial convergence** applied to sites where remote sensing and local knowledge agreed directionally but community consensus was weaker (between 35 and 65 percent), or where responses were mixed and no single category exceeded 65 percent. In Hlanzoun (Benin), remote sensing showed a slight net decrease (568.4 ha to 548.6 ha), while local reports were divided: 46% of respondents observed forest increase and 35% reported decline. This heterogeneity, falling within the 35–65% agreement range, indicates that community members experienced forest change differently, with no single narrative dominating. In Koui (Togo) remote sensing detected minimal forest loss (130.9 ha to 125.6 ha), While 54% of respondents reported regrowth linked to stricter governance. However, nearly one-third still reported decline, placing the site within the partial convergence category.

**Dissonance** was evident in sites where remote sensing and local knowledge were in clear contradiction, with at least 65% of respondents reporting a direction opposite to the satellite signal. In Ngam-Kondomeyos (Cameroon), forest cover remained **stable**—defined as less than 2% net change from 2010 to 2022 with no significant linear trend (p ≥ 0.10)—moving only slightly from 1285.0 ha to 1283.9 ha (a 0.09% net loss). Yet, 95% of respondents described decline attributed to selective logging and NTFP collection. A similar situation was observed in Mbangassina (Cameroon), where forest cover was effectively stable (1007.0 ha to 991.2 ha, a 1.6% net loss), but 94% of respondents reported forest decline, pointing to degradation processes such as selective logging and understory clearance that are not well captured by 30 m resolution data. Ikot (Nigeria) also fell within the stability threshold (1270.7 ha to 1253.0 ha, a 1.4% net loss), while 65% of respondents described significant degradation linked to logging, clearing for construction, and NTFP extraction. Together, these cases highlight the limitations of medium-resolution remote sensing in detecting subtle but socially and ecologically significant forms of degradation that communities experience firsthand.

Across all sites, signals of forest regrowth in the remote sensing data were limited. No forest patch exhibited strong or sustained increases in forest cover between 2010 and 2022. Consequently, there were no systematic instances where satellite-detected regrowth conflicted with local reports. Where regrowth was reported by communities, such as in Koui or Hlanzoun, it was typically attributed to factors like access restrictions or natural ecological recovery, and was either corroborated by the satellite data or fell below the threshold of detection.

Overall, the convergence matrix highlighted both corroborated trends and areas of divergence, providing a structured lens through which to interpret complex land-use dynamics. These findings underscore the complementary strengths of remote sensing and participatory methods in capturing multiscale forest change processes across diverse social-ecological contexts in West Africa.

## Discussion

Understanding forest change in tropical landscapes requires more than detecting vegetation loss; it necessitates engaging with the diverse ways in which communities experience, interpret, and respond to environmental transformations^18,19^. Our comparative analysis of Landsat-based forest cover trends and local knowledge across nine forest patches in West Africa advances this agenda by revealing zones of convergence and divergence between remote sensing data and community-reported insights. These patterns are not simply empirical findings but constitute epistemological signals, inviting reflection on how knowledge is produced, for whom, and with what implications for governance and justice.

In areas of full convergence, such as Agou and Elavagnon-Todji (Togo), Ewè-Adakplamè (Benin), and Iko (Nigeria), remotely sensed deforestation converged with local accounts of degradation, driven primarily by logging, slash-and-burn agriculture, and infrastructural expansion. These results affirm the utility of medium-resolution satellite imagery in capturing broad forest loss trends and are consistent with regional studies identifying similar proximate drivers^20–23^. However, in other sites, particularly Mbangassina and Ngam-Kondomeyos in Cameroon as well as Ikot in Nigeria, substantial community-reported degradation was not reflected in satellite-derived forest metrics. These cases of divergence are analytically productive, revealing the limitations of canopy-cover thresholds and satellite-based change detection when faced with subtle yet ecologically and socially significant processes such as canopy thinning, understory clearance, and resource extraction^9,24^.

Such divergences point to a broader and persistent challenge of scale mismatch in socio-ecological systems research^25,26^. Remote sensing approaches rely on standardized definitions of forest—typically ≥ 0.5 ha in area, ≥ 5 m in tree height, and ≥ 10% canopy cover^27^—that enable global comparability but may overlook the nuanced and situated ways in which local communities define and engage with forested landscapes. Local conceptualizations are often more expansive and qualitative, encompassing not only canopy density but also species richness, understory integrity, resource availability (e.g., firewood, medicinal plants), and accessibility^28,29^. In designing our household surveys, we explicitly privileged these local framings to improve ecological and cultural validity, accepting the analytical tensions that arise when distinct knowledge systems operate on different spatial and thematic registers.

Temporal mismatches added another layer of interpretive complexity. Some respondents may have described degradation processes or events that predate the satellite observation window used in our convergence analysis (2010–2022). Although our remote sensing dataset extends back to 2000 to provide a broader historical context, our comparative analysis was deliberately restricted to the post-2010 period to align with the ten-year (2012–2022) recall structure of the household surveys. To further balance historical depth with recall reliability, we also included a five-year recall horizon, which helped capture more recent, fine-grained dynamics while reducing memory bias^30^. Nonetheless, it is plausible that local knowledge of forest change drew on longer-term environmental memory, particularly in areas where degradation has been cumulative or episodic. Similar patterns have been observed in other regions where Indigenous and local ecological knowledge systems encode landscape histories that exceed the temporal reach of satellite archives^14^. These forms of temporal disjuncture reveal not only methodological limitations but also the fundamentally different ways in which communities construct environmental baselines and evaluate change^31^.

Beyond these temporal disjunctures, spatial resolution constraints further underscore the limitations of medium-resolution satellite imagery in capturing forest degradation. While the Landsat archive offers unmatched temporal depth (dating back to the 1970s), its 30 m spatial resolution is often insufficient to detect fine-scale processes such as canopy thinning, selective logging, understory removal, or fuelwood extraction—activities that may have high ecological and social salience but remain spectrally subtle^8,9,32^. Newer high-resolution platforms, such as PlanetScope (~ 3 m) and WorldView (~ 0.3 m), have enhanced capacity to capture such dynamics^33^, but lack the longitudinal consistency necessary to trace changes across decades. A promising way forward lies in multi-sensor fusion approaches that combine Landsat’s historical depth with the spatial granularity of newer sensors^34,35^. However, technical advances alone are insufficient. As critical remote sensing scholarship reminds us, sensor choices and classification schemes carry political effects. Co-designing indicators with communities, communicating uncertainty, and safeguarding sensitive places must therefore accompany multi-sensor integration^36,37^. Accordingly, embedding these systems within participatory and ethnographic frameworks, where local knowledge, values, and observations are systematically incorporated, can help ensure that forest condition assessments becomes not only more accurate, but also more socially responsive and epistemologically inclusive^38,39^.

At the same time, our findings show that participatory data are shaped by more than observational uncertainty. The high proportion of “nothing to report” responses in Ewè-Adakplamè and Ikot, for instance, may reflect hesitancy to speak on contested issues, fear of repercussions, or local norms discouraging attribution of blame in contexts of sensitive land tenure and resource access^40,41^. One respondent in Ewè-Adakplamè explicitly attributed forest loss to local conflict, suggesting that silence may mask underlying disputes. Beyond silence, deliberate misreporting is also possible, particularly where logging or NTFP harvesting intersects with contested livelihoods or governance arrangements. In such contexts, respondents may strategically downplay or exaggerate forest change depending on whether disclosure aligns with household, communal, or external interests. These dynamics underscore the need for caution when interpreting non-response or antagonistic accounts, and the importance of triangulating participatory data with contextual political–ecological knowledge.

Social representativeness is another limitation. Gendered patterns of participation, such as the dominance of male respondents in some sites (e.g., 93% in Ikot), raise concerns about whose knowledge is captured. Women’s roles in fuelwood collection, medicinal plant use, and smallholder agriculture often position them as key observers of forest change, yet their perspectives are systematically underrepresented in standard household surveys that target household heads, who are predominantly male^42,43^. Methodologically, this calls for a more inclusive design that incorporates women-specific focus groups, oral histories, or participatory mapping in order to capture a fuller range of environmental knowledge.

Local knowledge is also spatially heterogeneous. In Agou, Hlanzoun, and Ikot, for example, accounts of forest change varied significantly between sub-communities (p < 0.05), despite shared proximity to the same forest patches. This heterogeneity likely reflects micro-variations in forest access, livelihood dependence, and land-use histories, reinforcing the importance of fine-grained ethnographic and participatory assessments^44–46^. Although our stratified sample of 2,621 households enhances generalizability, scale mismatches between household experience and patch-level satellite analysis remain an inherent limitation.

More broadly, while our study identifies socio-demographic correlates of local knowledge, it does not fully capture the structural dynamics that shape forest use and cover change. Processes such as land tenure insecurity, customary authority, market integration, and shifting governance arrangements mediate how communities interact with forests and influence whether landscapes move toward degradation or regrowth^47–49^. Proximate drivers like population growth or agricultural expansion are therefore best understood as expressions of these deeper institutional and political-economic contexts, which also include policy reforms, logging concessions, and cross-scale power relations regulating access and control^50^. Demographic variables remain valuable for explaining variation in reported knowledge, but they cannot substitute for analysis of the institutional and governance dimensions of forest change. A promising next step is to integrate household-level surveys with participatory governance assessments and political–ecological approaches, enabling a clearer connection between observed forest change and the underlying social and institutional processes.

In light of these findings, our use of a convergence matrix offers more than a descriptive tool; it provides a framework for identifying synergies and tensions between remote sensing outputs and community knowledge. By classifying sites according to directional agreement across data types, the matrix highlights where scientific and experiential knowledge converge, and where further interpretive work is needed to reconcile them. Such approaches resonate with recent calls for knowledge co-production and transformative adaptation in sustainability science, which emphasize frameworks that value plural, negotiated understandings of environmental change^12,51^, while acknowledging the limits of technical tools in addressing deeper issues of epistemic justice and coloniality^52^.

Crucially, our findings challenge the assumption that divergence between satellite data and local accounts reflects error. Instead, these divergences reveal diagnostic complexity, pointing to ecological changes invisible to dominant remote sensing tools. For instance, in Mbangassina, residents described edge degradation and gradual canopy thinning—subtle but important processes undetected by 30 m Landsat imagery. Such observations demonstrate how small-scale practices accumulate into broader impacts over time. As emphasized in prior work^14^, addressing these gaps requires integrative approaches that bridge ways of knowing, combining local observations with biophysical data for a more comprehensive understanding of environmental change.

This integrative approach has important implications for global monitoring initiatives such as GFW and MRV-REDD + , which increasingly rely on satellite-derived data for policy, finance, and compliance decisions. While our study is best described as a forest condition assessment, its framework illustrates how multi-scalar analyses can inform more inclusive and adaptive monitoring systems. Incorporating local knowledge into MRV is not only an ethical matter of justice but also a strategic necessity for ensuring adaptive and accountable governance. As highlighted in prior research, when participation is absent or superficial, MRV risks becoming a technocratic exercise that sidelines the knowledge, needs, and rights of forest-dependent communities^53–55^. Building participatory systems requires sustained engagement with local communities, explicit attention to ethical participation, and mechanisms ensuring that monitoring outcomes deliver tangible benefits. Without these commitments, global monitoring programs risk misdiagnosing ecological trajectories, overlooking subtle degradation processes, or alienating the very communities whose cooperation is essential to durable stewardship^55–57^. Considering local knowledge is therefore both an ethical imperative and a governance strategy that strengthens the legitimacy and effectiveness of forest policy.

Our study shows that integrating remote sensing with local knowledge is not only about improving data validity. It clarifies where both approaches converge and where they diverge, particularly for degradation processes that are ecologically and socially significant but difficult to capture at medium resolution. This dual perspective redefines what counts as evidence, highlights the diagnostic value of divergences, and challenges extractive models of environmental knowledge production. It also underscores the need for monitoring frameworks that are both technically robust and socially inclusive. As new technologies generate increasingly fine-grained imagery, the priority is not only to sharpen spatial detail but also to integrate diverse ways of seeing, knowing, and living with forests. Such integration strengthens the scientific basis for monitoring while fostering conservation approaches that are more equitable, context-sensitive, and responsive to local realities.

## Methods

### Remote sensing and forest cover change analysis

We examined forest cover dynamics across nine focal forest patches located in Togo, Benin, Nigeria, and Cameroon, spanning humid (1,500–3,000 mm annual rainfall) to subhumid (600–1,000 mm) agroecological zones (Supplementary Fig. S1). These sites capture the region’s ecological diversity—from coastal plains with mangrove forests to inland landscapes of tropical moist forests and woodland savannas^58,59^. Annual mean temperatures range between 20–30 °C, while rainfall exhibits strong latitudinal gradients, with bimodal precipitation patterns in southern coastal areas and a single rainy season in the north^60^.

To assess forest cover and change from 2000 to 2022, we used Landsat 5 TM, 7 ETM + , and 8 OLI imagery (30 m resolution) available via GEE^61^. Peak-growing-season (June–September) cloud-free image composites were generated every five years (2000, 2005, 2010, 2015, 2020), and 2022 for recent conditions^62^. Preprocessing involved atmospheric correction^63^, shadow masking with the *FMask* algorithm^64^, and terrain corrections using the Shuttle Radar Topography Mission (SRTM) digital elevation model^65^.

We defined forest as areas ≥ 0.5 ha with ≥ 5 m tree height and ≥ 10% canopy cover^27^, employing a Random Forest (RF) classifier to distinguish forest from other land-use classes such as cropland, shrubland, sparse vegetation, wetland, waterbody, and built-up areas^66^. To support the ≥ 5 m height threshold, we used canopy height estimates from NASA’s Global Ecosystem Dynamics Investigation (GEDI) mission to guide training data selection and classifier calibration^67,68^. The classification accuracy was enhanced by incorporating spectral indices as independent bands to the original spectral band images^69^. These included the Normalized Difference Built-up Index (NDBI), Normalized Difference Vegetation Index (NDVI), Normalized Difference Water Index (NDWI), and Enhanced Vegetation Index (EVI), as defined in Eqs. 1 to 4, The RF classifier was trained using labelled data derived from 2022 imagery and consistently applied across the time series to ensure temporal comparability.

1 $$NDBI =(SWIR - NIR)/(SWIR + NIR)$$

2 $$NDVI = (NIR - Red)/(NIR + Red)$$

3 $$NDWI = (Green - NIR)/(Green + NIR)$$

4 $$EVI = G \times [(NIR - Red)/(NIR + C1 \times Red - C2 \times Blue + L)]$$

where NIR and SWIR refer to the near-infrared and short-wave infrared bands, respectively; Red and Green denote the red and green spectral bands. G represents a gain factor (commonly set to 2.5), while C1 and C2 are atmospheric resistance coefficients (typically 6 and 7.5). L is the soil adjustment factor, usually set to 1.

To detect spatiotemporal patterns of forest change, we applied a post-classification change detection approach^5^, comparing land cover classifications derived from Landsat imagery at the six time points (2000, 2005, 2010, 2015, 2020, 2022). Forest area within each patch was extracted from classified maps and expressed annually as a percentage relative to its 2000 baseline extent (set to 100%). This enabled consistent comparison of change trajectories across diverse ecological contexts. Linear trend models were fitted to these normalized time series to quantify the direction and magnitude of forest change over the 22-year period. At the national scale, forest area was similarly aggregated within administrative boundaries for Togo, Benin, Nigeria, and Cameroon to derive country-level trends.

To complement these temporal assessments, we tracked land cover transitions by intersecting classified maps from sequential years to identify pixel-level shifts between land cover classes. Changes in the forest class were used to detect forest loss, regrowth, and stability over time. For each forest patch and time interval, we generated land cover transition matrices to capture shifts from forest to cropland, shrubland, or other categories. These transitions were compiled into a consolidated database and visualized using Sankey diagrams to illustrate the direction, magnitude, and timing of key land use changes. These analyses provide a multi-scalar view of forest dynamics across local and national scales from 2000 to 2022.

Two independent accuracy assessments were carried out, each based on 1,500 reference sample points selected by stratified random sampling across the study area^70^. Every sample was visually interpreted on high-resolution Google Earth imagery (≤ 3 m) and, where possible, corroborated by field observations. The first assessment evaluated the seven-class land-use/land-cover map (Supplementary Table 5), whereas the second evaluated the three-class forest-change map produced by post-classification comparison (Supplementary Table 6). For both assessments we report overall accuracy as well as producer’s and user’s accuracies with binomial standard errors^71^.

### Household surveys and socio-economic data collection

Between October 2022 and March 2023, we conducted 2,621 semi-structured in-depth household surveys in communities located within 10 km of the nine forest patch sites. A stratified random sampling strategy was used to ensure representation across diverse settlements around the forests. To align community responses with the forest patches analyzed through remote sensing, survey questions were explicitly anchored to the specific patch assessed by satellite, using maps, village discussions, and locally recognized forest names as reference points.

The surveys focused on two themes: (i) local knowledge of changes in forest cover, and (ii) relevant socio-economic and institutional drivers over five-year and ten-year recall periods. We used both recall horizons to balance short-term accuracy with longer-term perspective. The 5-year period helped reduce recall bias and capture recent, fine-grained dynamics, while the 10-year period enabled respondents to draw on deeper environmental memory and report cumulative or episodic changes across a broader timescale.

Surveys were administered face-to-face by trained local enumerators fluent in French, English, and the respondents’ local language to ensure effective communication and accurate data collection. Informed consent was obtained from all participants prior to survey administration. Data were collected digitally using tablets with EpiCollect5™ software, streamlining data entry and minimizing transcription errors. Prior to implementation, the questionnaire was piloted in a comparable community to refine questions and format for clarity and cultural relevance. As part of this process, key terms describing forest processes (e.g., “forest cover change”) were carefully translated into local languages and refined through consultations with community leaders and partners, ensuring that remote sensing categories were understood in ways consistent with local interpretations.

Survey data were processed to address missing values, outliers, and inconsistencies. Descriptive statistical analyses were conducted in R software^72^ to summarize key variables such as demographics, livelihoods, and local knowledge of forest change. Because the data were not normally distributed, non-parametric inferential statistical tests were used to examine relationships between variables. Specifically, we used Fisher’s Exact Test for categorical variables with few levels (e.g., gender, marital status, and age) and the Kruskal–Wallis H test for variables with multiple levels (e.g., community and education level). This analysis enabled us to examine the relationships between socio-demographic variables and reported local knowledge of forest change.

### Data integration and convergence analysis

We employed an adapted convergence matrix framework^73^ to synthesize remote sensing outputs with household survey findings. This comparative approach compared two thematic dimensions: (1) forest cover change, and (2) evidence of regrowth across data sources. To assess agreement between data sources, we classified each forest patch into one of three categories based on both directional alignment and the proportion of community responses.

- **Full convergence** was assigned when remote sensing and local knowledge indicated the same direction of change, with 65% or more of community responses supporting that direction. For example, in Ewè-Adakplamè, 93% of respondents reported forest decline, consistent with satellite-detected loss.
- **Partial convergence** applied when the direction of change was consistent across data sources but community consensus was weaker (between 35 and 65%), or when responses were mixed and no single category exceeded 65 percent. In Hlanzoun, for instance, remote sensing showed slight forest loss, while 46% of respondents reported increase and 35% reported decline, indicating heterogeneous responses.
- **Dissonance** was used when remote sensing and local knowledge were in clear contradiction, specifically when 65% or more of community responses pointed in the opposite direction of the satellite signal. In Ngam-Kondomeyos, remote sensing indicated forest stability, yet 95% of respondents reported decline, suggesting degradation processes not captured by medium-resolution imagery.

To ensure consistent classification, thresholds were applied strictly: full convergence required 65% or more agreement in the same direction, partial convergence required 35 to 65% agreement or mixed responses, and dissonance required at least 65% of responses contradicting the remote sensing signal. Where disagreement arose, we consulted supplementary data sources, including Google Earth’s historical imagery archives and high-resolution drone imagery (< 10 cm spatial resolution, collected in 2022) to clarify small-scale disturbances such as canopy thinning, understory clearance, and edge degradation that may fall below the detection threshold of 30-m satellite imagery. This triangulation identified the extent to which socio-economic realities either corroborated or contrasted with pixel-based analyses, thereby enabling a holistic interpretation of forest change processes.

### Ethical considerations

All field protocols followed international guidelines for social research ethics^74^. We obtained research ethics approval from the Institute of Geography, University of Bern and country-specific research permits in Togo, Benin, Nigeria, and Cameroon. Enumerators provided study details and informed consent forms in local languages, emphasizing voluntary participation and the right to withdraw at any time. Informed consent was obtained from all participants prior to data collection. Participant data was kept confidential using coded identifiers and secure data-storage protocols. Throughout the field activities, we respected local norms and engaged community leaders to ensure culturally sensitive interactions. All methods were carried out in accordance with relevant guidelines and regulations.

## Supplementary Information

Supplementary Information.

## Data Availability

The remote sensing data that support the findings of this study are available from the corresponding author upon reasonable request. The household survey data contain sensitive personal information from participants and cannot be made publicly available due to privacy concerns and ethical restrictions for protecting respondent confidentiality.

## Code availability

The R and Python scripts used in this study are available in a public GitHub repository: https://github.com/ciheaturu/remote-sensing-local-narratives/tree/main.
