# Supplementary material for: Combining remote sensing with local knowledge is vital for understanding forest change in West Africa
Source: Sci Rep. 2025 Oct 30;15:38094. doi: 10.1038/s41598-025-23133-5 (PMC12575867; doi:10.1038/s41598-025-23133-5)
Supplement: Supplementary file 1 — Supplementary Information. [file 41598_2025_23133_MOESM1_ESM.docx]

**Supplementary Information: Combining remote sensing with local knowledge is vital for understanding forest change in West Africa**

**Supplementary Table 1. Socio-ecological characteristics of nine focal forest patches in West Africa.** For each patch in Togo, Benin, Nigeria and Cameroon, the table lists the governing tenure regime (community versus mixed community–family ownership), neighboring settlements that depend on or manage the forest, and the dominant agro-ecological zone classified as humid-warm or sub-humid-warm.

| **Country** | **Forest patch** | **Tenure** | **Community** | **Agroecological zone** |
| --- | --- | --- | --- | --- |
| Togo | Agou | Community and Family (Mixed) | Akplolo | Subhumid warm |
|  |  |  | Kébo Djigbé |  |
|  |  |  | Kébo Dogbadji |  |
|  |  |  | Kébo Domépémé |  |
|  |  |  | Kébo Etui |  |
|  |  |  | Kébo Kpéta |  |
|  |  |  | Kébo Apégamé |  |
|  |  |  | Kébo Tomégbé |  |
|  | Elavagnon-Todji | Community and Family (Mixed) | Elavagnon-Todji | Subhumid warm |
|  |  |  | M’poti |  |
|  | Koui | Community | Koui | Subhumid warm |
| Benin | Ewè-Adakplamè | Community | Adakplamè | Subhumid warm |
|  |  |  | Edenou |  |
|  |  |  | Ewè |  |
|  | Hlanzoun (AKA Lokoli) | Community | Dèmè | Subhumid warm |
|  |  |  | Koussoukpa |  |
|  |  |  | Lokoli |  |
| Nigeria | Iko | Community | Iko Ekperem | Humid warm |
|  |  |  | Iko Esai |  |
|  |  |  | Owai |  |
|  | Ikot | Community and Family (Mixed) | Ikot Abia | Humid warm |
|  |  |  | Ikot Akpandem |  |
|  |  |  | lkot Ataku |  |
|  |  |  | Ikot Inyang |  |
|  |  |  | Ikot Obioro |  |
|  |  |  | Okon Eket |  |
| Cameroon | Mbangassina | Community and Family (Mixed) | Ebina | Humid warm |
|  |  |  | Etam Nyat |  |
|  |  |  | Mbianga |  |
|  |  |  | Teate |  |
|  | Ngam-Kondomeyos | Community and Family (Mixed) | Ngam | Humid warm |
|  |  |  | Kondomeyos |  |

**Supplementary Table 2. Temporal trends in forest extent for nine focal forest patches (2000–2022).** Area of forest (hectares) derived from Landsat-based classifications (30 m resolution) at six observation years. Forest is defined as contiguous canopy ≥ 0.5 ha, height ≥ 5 m and canopy cover ≥ 10 %. See Methods for classification workflow and accuracy assessment.

| **Forest patch** | **Area (ha)** | | | | | |
| --- | --- | --- | --- | --- | --- | --- |
|  | **2000** | **2005** | **2010** | **2015** | **2020** | **2022** |
| Agou | 2355.12 | 2346.12 | 2346.03 | 2327.22 | 2281.68 | 2280.85 |
| Elavagnon-Todji | 697.77 | 691.56 | 680.76 | 662.31 | 649.8 | 648.41 |
| Koui | 133.2 | 130.86 | 130.95 | 129.6 | 126.63 | 125.63 |
| Ewè-Adakplamè | 619.2 | 594.72 | 586.89 | 573.48 | 528.84 | 505.42 |
| Hlanzoun | 547.38 | 558.18 | 568.44 | 577.44 | 559.26 | 548.61 |
| Iko | 3557.7 | 3559.23 | 3554.19 | 3555.63 | 3428.64 | 3423.52 |
| Ikot | 1270.8 | 1271.7 | 1270.71 | 1254.51 | 1253.07 | 1252.97 |
| Mbangassina | 1012.23 | 1009.98 | 1007.01 | 997.83 | 993.15 | 991.23 |
| Ngam-Kondomeyos | 1283.58 | 1285.02 | 1285.02 | 1284.93 | 1284.84 | 1283.9 |

**Supplementary Table 3. Land-cover transitions detected in nine West African forest patches between 2000 and 2022.** For each patch, the table lists the area (hectares) that shifted from an initial land-cover class (“From”) to a subsequent class (“To”) over five consecutive intervals (2000–2005, 2005–2010, 2010–2015, 2015–2020, 2020–2022). Values are derived from pixel-by-pixel post-classification change detection of Landsat imagery (30 m resolution). Only transitions ≥0.09 ha (one Landsat pixel) are shown.

| **Patch** | **Year from** | **Year to** | **From** | **To** | **Hectares** |
| --- | --- | --- | --- | --- | --- |
| Agou | 2000 | 2005 | Shrubland | Shrubland | 131.49 |
| Agou | 2000 | 2005 | Shrubland | Forest | 6.93 |
| Agou | 2000 | 2005 | Shrubland | Builtup area | 0.9 |
| Agou | 2000 | 2005 | Forest | Shrubland | 12.96 |
| Agou | 2000 | 2005 | Forest | Forest | 2339.19 |
| Agou | 2000 | 2005 | Forest | Builtup area | 2.97 |
| Agou | 2000 | 2005 | Builtup area | Builtup area | 58.23 |
| Agou | 2005 | 2010 | Shrubland | Shrubland | 129.69 |
| Agou | 2005 | 2010 | Shrubland | Forest | 12.42 |
| Agou | 2005 | 2010 | Shrubland | Builtup area | 2.34 |
| Agou | 2005 | 2010 | Forest | Shrubland | 8.64 |
| Agou | 2005 | 2010 | Forest | Forest | 2333.61 |
| Agou | 2005 | 2010 | Forest | Builtup area | 3.87 |
| Agou | 2005 | 2010 | Builtup area | Builtup area | 62.1 |
| Agou | 2010 | 2015 | Shrubland | Shrubland | 127.62 |
| Agou | 2010 | 2015 | Shrubland | Forest | 10.08 |
| Agou | 2010 | 2015 | Shrubland | Builtup area | 0.63 |
| Agou | 2010 | 2015 | Forest | Shrubland | 21.69 |
| Agou | 2010 | 2015 | Forest | Forest | 2317.14 |
| Agou | 2010 | 2015 | Forest | Builtup area | 7.2 |
| Agou | 2010 | 2015 | Builtup area | Builtup area | 68.31 |
| Agou | 2015 | 2020 | Shrubland | Shrubland | 137.07 |
| Agou | 2015 | 2020 | Shrubland | Forest | 9.81 |
| Agou | 2015 | 2020 | Shrubland | Builtup area | 2.43 |
| Agou | 2015 | 2020 | Forest | Shrubland | 40.77 |
| Agou | 2015 | 2020 | Forest | Forest | 2271.87 |
| Agou | 2015 | 2020 | Forest | Builtup area | 14.58 |
| Agou | 2015 | 2020 | Builtup area | Builtup area | 76.14 |
| Agou | 2020 | 2022 | Shrubland | Shrubland | 177.84 |
| Agou | 2020 | 2022 | Forest | Forest | 2280.85 |
| Agou | 2020 | 2022 | Forest | Shrubland | 0.83 |
| Agou | 2020 | 2022 | Builtup area | Builtup area | 93.15 |
| Elavagnon-Todji | 2000 | 2005 | Shrubland | Shrubland | 93.24 |
| Elavagnon-Todji | 2000 | 2005 | Shrubland | Forest | 0.81 |
| Elavagnon-Todji | 2000 | 2005 | Shrubland | Builtup area | 0.09 |
| Elavagnon-Todji | 2000 | 2005 | Forest | Shrubland | 6.39 |
| Elavagnon-Todji | 2000 | 2005 | Forest | Forest | 690.75 |
| Elavagnon-Todji | 2000 | 2005 | Forest | Builtup area | 0.63 |
| Elavagnon-Todji | 2000 | 2005 | Builtup area | Builtup area | 5.22 |
| Elavagnon-Todji | 2005 | 2010 | Shrubland | Shrubland | 94.77 |
| Elavagnon-Todji | 2005 | 2010 | Shrubland | Forest | 4.41 |
| Elavagnon-Todji | 2005 | 2010 | Shrubland | Builtup area | 0.45 |
| Elavagnon-Todji | 2005 | 2010 | Forest | Shrubland | 13.68 |
| Elavagnon-Todji | 2005 | 2010 | Forest | Forest | 676.35 |
| Elavagnon-Todji | 2005 | 2010 | Forest | Builtup area | 1.53 |
| Elavagnon-Todji | 2005 | 2010 | Builtup area | Builtup area | 5.94 |
| Elavagnon-Todji | 2010 | 2015 | Shrubland | Shrubland | 98.46 |
| Elavagnon-Todji | 2010 | 2015 | Shrubland | Forest | 9.54 |
| Elavagnon-Todji | 2010 | 2015 | Shrubland | Builtup area | 0.45 |
| Elavagnon-Todji | 2010 | 2015 | Forest | Shrubland | 27.09 |
| Elavagnon-Todji | 2010 | 2015 | Forest | Forest | 652.77 |
| Elavagnon-Todji | 2010 | 2015 | Forest | Builtup area | 0.9 |
| Elavagnon-Todji | 2010 | 2015 | Builtup area | Builtup area | 7.92 |
| Elavagnon-Todji | 2015 | 2020 | Shrubland | Shrubland | 107.28 |
| Elavagnon-Todji | 2015 | 2020 | Shrubland | Forest | 18.18 |
| Elavagnon-Todji | 2015 | 2020 | Shrubland | Builtup area | 0.09 |
| Elavagnon-Todji | 2015 | 2020 | Forest | Shrubland | 28.98 |
| Elavagnon-Todji | 2015 | 2020 | Forest | Forest | 631.62 |
| Elavagnon-Todji | 2015 | 2020 | Forest | Builtup area | 1.71 |
| Elavagnon-Todji | 2015 | 2020 | Builtup area | Builtup area | 9.27 |
| Elavagnon-Todji | 2020 | 2022 | Shrubland | Shrubland | 136.26 |
| Elavagnon-Todji | 2020 | 2022 | Forest | Forest | 648.41 |
| Elavagnon-Todji | 2020 | 2022 | Forest | Shrubland | 1.39 |
| Elavagnon-Todji | 2020 | 2022 | Builtup area | Builtup area | 11.07 |
| Koui | 2000 | 2005 | Shrubland | Shrubland | 22.77 |
| Koui | 2000 | 2005 | Shrubland | Forest | 0.27 |
| Koui | 2000 | 2005 | Shrubland | Builtup area | 2.43 |
| Koui | 2000 | 2005 | Forest | Shrubland | 0.45 |
| Koui | 2000 | 2005 | Forest | Forest | 130.59 |
| Koui | 2000 | 2005 | Forest | Builtup area | 2.16 |
| Koui | 2000 | 2005 | Builtup area | Builtup area | 7.74 |
| Koui | 2005 | 2010 | Shrubland | Shrubland | 22.95 |
| Koui | 2005 | 2010 | Shrubland | Forest | 0.27 |
| Koui | 2005 | 2010 | Forest | Shrubland | 0.09 |
| Koui | 2005 | 2010 | Forest | Forest | 130.68 |
| Koui | 2005 | 2010 | Forest | Builtup area | 0.09 |
| Koui | 2005 | 2010 | Builtup area | Builtup area | 12.33 |
| Koui | 2010 | 2015 | Shrubland | Shrubland | 21.24 |
| Koui | 2010 | 2015 | Shrubland | Forest | 0.27 |
| Koui | 2010 | 2015 | Shrubland | Builtup area | 1.53 |
| Koui | 2010 | 2015 | Forest | Shrubland | 0.18 |
| Koui | 2010 | 2015 | Forest | Forest | 129.33 |
| Koui | 2010 | 2015 | Forest | Builtup area | 1.44 |
| Koui | 2010 | 2015 | Builtup area | Builtup area | 12.42 |
| Koui | 2015 | 2020 | Shrubland | Shrubland | 20.97 |
| Koui | 2015 | 2020 | Shrubland | Forest | 0.09 |
| Koui | 2015 | 2020 | Shrubland | Builtup area | 0.36 |
| Koui | 2015 | 2020 | Forest | Shrubland | 2.34 |
| Koui | 2015 | 2020 | Forest | Forest | 126.54 |
| Koui | 2015 | 2020 | Forest | Builtup area | 0.72 |
| Koui | 2015 | 2020 | Builtup area | Builtup area | 15.39 |
| Koui | 2020 | 2022 | Shrubland | Shrubland | 23.31 |
| Koui | 2020 | 2022 | Forest | Forest | 125.63 |
| Koui | 2020 | 2022 | Forest | Shrubland | 1 |
| Koui | 2020 | 2022 | Builtup area | Builtup area | 16.47 |
| Ewè-Adakplamè | 2000 | 2005 | Shrubland | Shrubland | 728.64 |
| Ewè-Adakplamè | 2000 | 2005 | Shrubland | Forest | 1.17 |
| Ewè-Adakplamè | 2000 | 2005 | Shrubland | Cropland | 102.78 |
| Ewè-Adakplamè | 2000 | 2005 | Shrubland | Builtup area | 8.82 |
| Ewè-Adakplamè | 2000 | 2005 | Forest | Shrubland | 18.72 |
| Ewè-Adakplamè | 2000 | 2005 | Forest | Forest | 591.84 |
| Ewè-Adakplamè | 2000 | 2005 | Forest | Cropland | 6.75 |
| Ewè-Adakplamè | 2000 | 2005 | Forest | Builtup area | 1.89 |
| Ewè-Adakplamè | 2000 | 2005 | Cropland | Shrubland | 48.42 |
| Ewè-Adakplamè | 2000 | 2005 | Cropland | Forest | 1.71 |
| Ewè-Adakplamè | 2000 | 2005 | Cropland | Cropland | 168.12 |
| Ewè-Adakplamè | 2000 | 2005 | Cropland | Builtup area | 27.18 |
| Ewè-Adakplamè | 2000 | 2005 | Builtup area | Builtup area | 105.3 |
| Ewè-Adakplamè | 2005 | 2010 | Shrubland | Shrubland | 711.63 |
| Ewè-Adakplamè | 2005 | 2010 | Shrubland | Forest | 7.65 |
| Ewè-Adakplamè | 2005 | 2010 | Shrubland | Cropland | 75.51 |
| Ewè-Adakplamè | 2005 | 2010 | Shrubland | Builtup area | 0.99 |
| Ewè-Adakplamè | 2005 | 2010 | Forest | Shrubland | 13.5 |
| Ewè-Adakplamè | 2005 | 2010 | Forest | Forest | 578.7 |
| Ewè-Adakplamè | 2005 | 2010 | Forest | Cropland | 2.25 |
| Ewè-Adakplamè | 2005 | 2010 | Forest | Builtup area | 0.27 |
| Ewè-Adakplamè | 2005 | 2010 | Cropland | Shrubland | 14.4 |
| Ewè-Adakplamè | 2005 | 2010 | Cropland | Forest | 0.54 |
| Ewè-Adakplamè | 2005 | 2010 | Cropland | Cropland | 255.96 |
| Ewè-Adakplamè | 2005 | 2010 | Cropland | Builtup area | 6.75 |
| Ewè-Adakplamè | 2005 | 2010 | Builtup area | Builtup area | 143.19 |
| Ewè-Adakplamè | 2010 | 2015 | Shrubland | Shrubland | 670.95 |
| Ewè-Adakplamè | 2010 | 2015 | Shrubland | Forest | 3.96 |
| Ewè-Adakplamè | 2010 | 2015 | Shrubland | Cropland | 52.11 |
| Ewè-Adakplamè | 2010 | 2015 | Shrubland | Builtup area | 12.51 |
| Ewè-Adakplamè | 2010 | 2015 | Forest | Shrubland | 15.84 |
| Ewè-Adakplamè | 2010 | 2015 | Forest | Forest | 566.91 |
| Ewè-Adakplamè | 2010 | 2015 | Forest | Cropland | 1.53 |
| Ewè-Adakplamè | 2010 | 2015 | Forest | Builtup area | 2.61 |
| Ewè-Adakplamè | 2010 | 2015 | Cropland | Shrubland | 24.84 |
| Ewè-Adakplamè | 2010 | 2015 | Cropland | Forest | 2.61 |
| Ewè-Adakplamè | 2010 | 2015 | Cropland | Cropland | 267.75 |
| Ewè-Adakplamè | 2010 | 2015 | Cropland | Builtup area | 38.52 |
| Ewè-Adakplamè | 2010 | 2015 | Builtup area | Builtup area | 151.2 |
| Ewè-Adakplamè | 2015 | 2020 | Shrubland | Shrubland | 599.67 |
| Ewè-Adakplamè | 2015 | 2020 | Shrubland | Forest | 6.75 |
| Ewè-Adakplamè | 2015 | 2020 | Shrubland | Cropland | 98.01 |
| Ewè-Adakplamè | 2015 | 2020 | Shrubland | Builtup area | 7.2 |
| Ewè-Adakplamè | 2015 | 2020 | Forest | Shrubland | 50.76 |
| Ewè-Adakplamè | 2015 | 2020 | Forest | Forest | 520.65 |
| Ewè-Adakplamè | 2015 | 2020 | Forest | Cropland | 1.44 |
| Ewè-Adakplamè | 2015 | 2020 | Forest | Builtup area | 0.63 |
| Ewè-Adakplamè | 2015 | 2020 | Cropland | Shrubland | 53.19 |
| Ewè-Adakplamè | 2015 | 2020 | Cropland | Forest | 1.44 |
| Ewè-Adakplamè | 2015 | 2020 | Cropland | Cropland | 241.2 |
| Ewè-Adakplamè | 2015 | 2020 | Cropland | Builtup area | 25.56 |
| Ewè-Adakplamè | 2015 | 2020 | Builtup area | Builtup area | 204.84 |
| Ewè-Adakplamè | 2020 | 2022 | Shrubland | Shrubland | 703.62 |
| Ewè-Adakplamè | 2020 | 2022 | Forest | Forest | 505.42 |
| Ewè-Adakplamè | 2020 | 2022 | Forest | Shrubland | 23.42 |
| Ewè-Adakplamè | 2020 | 2022 | Cropland | Cropland | 340.65 |
| Ewè-Adakplamè | 2020 | 2022 | Builtup area | Builtup area | 238.23 |
| Hlanzoun | 2000 | 2005 | Shrubland | Shrubland | 1818.18 |
| Hlanzoun | 2000 | 2005 | Shrubland | Forest | 1.17 |
| Hlanzoun | 2000 | 2005 | Shrubland | Cropland | 200.61 |
| Hlanzoun | 2000 | 2005 | Shrubland | Builtup area | 0.54 |
| Hlanzoun | 2000 | 2005 | Forest | Shrubland | 3.33 |
| Hlanzoun | 2000 | 2005 | Forest | Forest | 538.11 |
| Hlanzoun | 2000 | 2005 | Forest | Wetland | 5.76 |
| Hlanzoun | 2000 | 2005 | Forest | Cropland | 0.18 |
| Hlanzoun | 2000 | 2005 | Wetland | Forest | 18.54 |
| Hlanzoun | 2000 | 2005 | Wetland | Wetland | 605.7 |
| Hlanzoun | 2000 | 2005 | Wetland | Cropland | 1.26 |
| Hlanzoun | 2000 | 2005 | Cropland | Shrubland | 215.19 |
| Hlanzoun | 2000 | 2005 | Cropland | Forest | 0.36 |
| Hlanzoun | 2000 | 2005 | Cropland | Wetland | 8.91 |
| Hlanzoun | 2000 | 2005 | Cropland | Cropland | 1097.28 |
| Hlanzoun | 2000 | 2005 | Cropland | Builtup area | 1.53 |
| Hlanzoun | 2000 | 2005 | Builtup area | Builtup area | 22.86 |
| Hlanzoun | 2005 | 2010 | Shrubland | Shrubland | 1991.61 |
| Hlanzoun | 2005 | 2010 | Shrubland | Forest | 1.26 |
| Hlanzoun | 2005 | 2010 | Shrubland | Cropland | 43.83 |
| Hlanzoun | 2005 | 2010 | Forest | Shrubland | 3.69 |
| Hlanzoun | 2005 | 2010 | Forest | Forest | 548.64 |
| Hlanzoun | 2005 | 2010 | Forest | Wetland | 5.85 |
| Hlanzoun | 2005 | 2010 | Wetland | Forest | 18.54 |
| Hlanzoun | 2005 | 2010 | Wetland | Wetland | 600.66 |
| Hlanzoun | 2005 | 2010 | Wetland | Cropland | 1.17 |
| Hlanzoun | 2005 | 2010 | Cropland | Shrubland | 77.04 |
| Hlanzoun | 2005 | 2010 | Cropland | Wetland | 0.99 |
| Hlanzoun | 2005 | 2010 | Cropland | Cropland | 1221.3 |
| Hlanzoun | 2005 | 2010 | Builtup area | Builtup area | 24.93 |
| Hlanzoun | 2010 | 2015 | Shrubland | Shrubland | 1910.7 |
| Hlanzoun | 2010 | 2015 | Shrubland | Forest | 1.71 |
| Hlanzoun | 2010 | 2015 | Shrubland | Cropland | 154.44 |
| Hlanzoun | 2010 | 2015 | Shrubland | Builtup area | 5.49 |
| Hlanzoun | 2010 | 2015 | Forest | Shrubland | 12.42 |
| Hlanzoun | 2010 | 2015 | Forest | Forest | 553.59 |
| Hlanzoun | 2010 | 2015 | Forest | Wetland | 2.43 |
| Hlanzoun | 2010 | 2015 | Wetland | Forest | 21.96 |
| Hlanzoun | 2010 | 2015 | Wetland | Wetland | 578.88 |
| Hlanzoun | 2010 | 2015 | Wetland | Cropland | 6.66 |
| Hlanzoun | 2010 | 2015 | Cropland | Shrubland | 80.1 |
| Hlanzoun | 2010 | 2015 | Cropland | Forest | 0.18 |
| Hlanzoun | 2010 | 2015 | Cropland | Wetland | 0.45 |
| Hlanzoun | 2010 | 2015 | Cropland | Cropland | 1184.13 |
| Hlanzoun | 2010 | 2015 | Cropland | Builtup area | 1.44 |
| Hlanzoun | 2010 | 2015 | Builtup area | Builtup area | 24.93 |
| Hlanzoun | 2015 | 2020 | Shrubland | Shrubland | 1864.35 |
| Hlanzoun | 2015 | 2020 | Shrubland | Forest | 5.76 |
| Hlanzoun | 2015 | 2020 | Shrubland | Cropland | 129.15 |
| Hlanzoun | 2015 | 2020 | Shrubland | Builtup area | 3.96 |
| Hlanzoun | 2015 | 2020 | Forest | Shrubland | 12.15 |
| Hlanzoun | 2015 | 2020 | Forest | Forest | 550.26 |
| Hlanzoun | 2015 | 2020 | Forest | Wetland | 15.03 |
| Hlanzoun | 2015 | 2020 | Wetland | Forest | 3.24 |
| Hlanzoun | 2015 | 2020 | Wetland | Wetland | 573.03 |
| Hlanzoun | 2015 | 2020 | Wetland | Cropland | 5.4 |
| Hlanzoun | 2015 | 2020 | Wetland | Builtup area | 0.09 |
| Hlanzoun | 2015 | 2020 | Cropland | Shrubland | 276.21 |
| Hlanzoun | 2015 | 2020 | Cropland | Wetland | 1.71 |
| Hlanzoun | 2015 | 2020 | Cropland | Cropland | 1057.95 |
| Hlanzoun | 2015 | 2020 | Cropland | Builtup area | 9.36 |
| Hlanzoun | 2015 | 2020 | Builtup area | Builtup area | 31.86 |
| Hlanzoun | 2020 | 2022 | Shrubland | Shrubland | 2152.71 |
| Hlanzoun | 2020 | 2022 | Forest | Forest | 548.61 |
| Hlanzoun | 2020 | 2022 | Forest | Wetland | 10.65 |
| Hlanzoun | 2020 | 2022 | Wetland | Wetland | 589.77 |
| Hlanzoun | 2020 | 2022 | Cropland | Cropland | 1192.5 |
| Hlanzoun | 2020 | 2022 | Builtup area | Builtup area | 45.27 |
| Iko | 2000 | 2005 | Shrubland | Shrubland | 8.01 |
| Iko | 2000 | 2005 | Shrubland | Forest | 3.06 |
| Iko | 2000 | 2005 | Forest | Shrubland | 1.53 |
| Iko | 2000 | 2005 | Forest | Forest | 3556.17 |
| Iko | 2000 | 2005 | Cropland | Cropland | 0.09 |
| Iko | 2000 | 2005 | Builtup area | Builtup area | 6.93 |
| Iko | 2005 | 2010 | Shrubland | Shrubland | 6.75 |
| Iko | 2005 | 2010 | Shrubland | Forest | 2.79 |
| Iko | 2005 | 2010 | Forest | Shrubland | 7.83 |
| Iko | 2005 | 2010 | Forest | Forest | 3551.4 |
| Iko | 2005 | 2010 | Cropland | Cropland | 0.09 |
| Iko | 2005 | 2010 | Builtup area | Builtup area | 6.93 |
| Iko | 2010 | 2015 | Shrubland | Shrubland | 7.38 |
| Iko | 2010 | 2015 | Shrubland | Forest | 7.2 |
| Iko | 2010 | 2015 | Forest | Shrubland | 5.76 |
| Iko | 2010 | 2015 | Forest | Forest | 3548.43 |
| Iko | 2010 | 2015 | Cropland | Cropland | 0.09 |
| Iko | 2010 | 2015 | Builtup area | Builtup area | 6.93 |
| Iko | 2015 | 2020 | Shrubland | Shrubland | 10.98 |
| Iko | 2015 | 2020 | Shrubland | Forest | 1.98 |
| Iko | 2015 | 2020 | Shrubland | Cropland | 0.09 |
| Iko | 2015 | 2020 | Shrubland | Builtup area | 0.09 |
| Iko | 2015 | 2020 | Forest | Shrubland | 128.7 |
| Iko | 2015 | 2020 | Forest | Forest | 3426.66 |
| Iko | 2015 | 2020 | Forest | Wetland | 0.09 |
| Iko | 2015 | 2020 | Forest | Builtup area | 0.18 |
| Iko | 2015 | 2020 | Cropland | Cropland | 0.09 |
| Iko | 2015 | 2020 | Builtup area | Builtup area | 6.93 |
| Iko | 2020 | 2022 | Shrubland | Shrubland | 139.68 |
| Iko | 2020 | 2022 | Forest | Forest | 3423.52 |
| Iko | 2020 | 2022 | Forest | Shrubland | 5.12 |
| Iko | 2020 | 2022 | Wetland | Wetland | 0.09 |
| Iko | 2020 | 2022 | Cropland | Cropland | 0.18 |
| Iko | 2020 | 2022 | Builtup area | Builtup area | 7.2 |
| Ikot | 2000 | 2005 | Shrubland | Shrubland | 258.39 |
| Ikot | 2000 | 2005 | Shrubland | Forest | 4.32 |
| Ikot | 2000 | 2005 | Shrubland | Waterbody | 0.99 |
| Ikot | 2000 | 2005 | Shrubland | Builtup area | 45.81 |
| Ikot | 2000 | 2005 | Forest | Shrubland | 0.54 |
| Ikot | 2000 | 2005 | Forest | Forest | 1264.14 |
| Ikot | 2000 | 2005 | Forest | Wetland | 0.45 |
| Ikot | 2000 | 2005 | Forest | Waterbody | 0.36 |
| Ikot | 2000 | 2005 | Forest | Builtup area | 5.31 |
| Ikot | 2000 | 2005 | Wetland | Forest | 2.79 |
| Ikot | 2000 | 2005 | Wetland | Wetland | 145.26 |
| Ikot | 2000 | 2005 | Wetland | Waterbody | 5.58 |
| Ikot | 2000 | 2005 | Wetland | Builtup area | 2.7 |
| Ikot | 2000 | 2005 | Waterbody | Shrubland | 0.9 |
| Ikot | 2000 | 2005 | Waterbody | Forest | 0.45 |
| Ikot | 2000 | 2005 | Waterbody | Wetland | 47.25 |
| Ikot | 2000 | 2005 | Waterbody | Waterbody | 145.35 |
| Ikot | 2000 | 2005 | Builtup area | Builtup area | 245.07 |
| Ikot | 2005 | 2010 | Shrubland | Shrubland | 221.4 |
| Ikot | 2005 | 2010 | Shrubland | Forest | 6.57 |
| Ikot | 2005 | 2010 | Shrubland | Waterbody | 1.35 |
| Ikot | 2005 | 2010 | Shrubland | Builtup area | 30.51 |
| Ikot | 2005 | 2010 | Forest | Shrubland | 2.34 |
| Ikot | 2005 | 2010 | Forest | Forest | 1262.52 |
| Ikot | 2005 | 2010 | Forest | Wetland | 1.08 |
| Ikot | 2005 | 2010 | Forest | Waterbody | 0.36 |
| Ikot | 2005 | 2010 | Forest | Builtup area | 5.4 |
| Ikot | 2005 | 2010 | Wetland | Forest | 1.53 |
| Ikot | 2005 | 2010 | Wetland | Wetland | 139.14 |
| Ikot | 2005 | 2010 | Wetland | Waterbody | 47.7 |
| Ikot | 2005 | 2010 | Wetland | Builtup area | 4.59 |
| Ikot | 2005 | 2010 | Waterbody | Sparse vegetation | 0.18 |
| Ikot | 2005 | 2010 | Waterbody | Shrubland | 1.08 |
| Ikot | 2005 | 2010 | Waterbody | Forest | 0.09 |
| Ikot | 2005 | 2010 | Waterbody | Wetland | 9.54 |
| Ikot | 2005 | 2010 | Waterbody | Waterbody | 140.94 |
| Ikot | 2005 | 2010 | Waterbody | Builtup area | 0.45 |
| Ikot | 2005 | 2010 | Builtup area | Builtup area | 298.89 |
| Ikot | 2010 | 2015 | Sparse vegetation | Waterbody | 0.18 |
| Ikot | 2010 | 2015 | Shrubland | Shrubland | 184.59 |
| Ikot | 2010 | 2015 | Shrubland | Forest | 1.53 |
| Ikot | 2010 | 2015 | Shrubland | Waterbody | 0.81 |
| Ikot | 2010 | 2015 | Shrubland | Builtup area | 37.89 |
| Ikot | 2010 | 2015 | Forest | Shrubland | 5.4 |
| Ikot | 2010 | 2015 | Forest | Forest | 1251.54 |
| Ikot | 2010 | 2015 | Forest | Wetland | 2.61 |
| Ikot | 2010 | 2015 | Forest | Waterbody | 0.09 |
| Ikot | 2010 | 2015 | Forest | Builtup area | 11.07 |
| Ikot | 2010 | 2015 | Wetland | Forest | 0.81 |
| Ikot | 2010 | 2015 | Wetland | Wetland | 124.38 |
| Ikot | 2010 | 2015 | Wetland | Waterbody | 10.08 |
| Ikot | 2010 | 2015 | Wetland | Builtup area | 14.49 |
| Ikot | 2010 | 2015 | Waterbody | Shrubland | 1.53 |
| Ikot | 2010 | 2015 | Waterbody | Forest | 0.63 |
| Ikot | 2010 | 2015 | Waterbody | Wetland | 17.55 |
| Ikot | 2010 | 2015 | Waterbody | Waterbody | 170.64 |
| Ikot | 2010 | 2015 | Builtup area | Builtup area | 339.84 |
| Ikot | 2015 | 2020 | Shrubland | Shrubland | 177.57 |
| Ikot | 2015 | 2020 | Shrubland | Forest | 2.97 |
| Ikot | 2015 | 2020 | Shrubland | Waterbody | 1.62 |
| Ikot | 2015 | 2020 | Shrubland | Builtup area | 9.36 |
| Ikot | 2015 | 2020 | Forest | Shrubland | 5.13 |
| Ikot | 2015 | 2020 | Forest | Forest | 1247.58 |
| Ikot | 2015 | 2020 | Forest | Wetland | 0.18 |
| Ikot | 2015 | 2020 | Forest | Waterbody | 0.36 |
| Ikot | 2015 | 2020 | Forest | Builtup area | 1.26 |
| Ikot | 2015 | 2020 | Wetland | Forest | 2.52 |
| Ikot | 2015 | 2020 | Wetland | Wetland | 120.24 |
| Ikot | 2015 | 2020 | Wetland | Waterbody | 12.87 |
| Ikot | 2015 | 2020 | Wetland | Builtup area | 8.91 |
| Ikot | 2015 | 2020 | Waterbody | Shrubland | 0.54 |
| Ikot | 2015 | 2020 | Waterbody | Wetland | 11.97 |
| Ikot | 2015 | 2020 | Waterbody | Waterbody | 169.29 |
| Ikot | 2015 | 2020 | Builtup area | Builtup area | 403.29 |
| Ikot | 2020 | 2022 | Shrubland | Shrubland | 183.24 |
| Ikot | 2020 | 2022 | Forest | Forest | 1252.97 |
| Ikot | 2020 | 2022 | Forest | Wetland | 0.1 |
| Ikot | 2020 | 2022 | Wetland | Wetland | 132.39 |
| Ikot | 2020 | 2022 | Waterbody | Waterbody | 184.14 |
| Ikot | 2020 | 2022 | Builtup area | Builtup area | 422.82 |
| Mbangassina | 2000 | 2005 | Shrubland | Shrubland | 11.16 |
| Mbangassina | 2000 | 2005 | Shrubland | Forest | 3.24 |
| Mbangassina | 2000 | 2005 | Shrubland | Builtup area | 0.99 |
| Mbangassina | 2000 | 2005 | Forest | Shrubland | 1.08 |
| Mbangassina | 2000 | 2005 | Forest | Forest | 1006.74 |
| Mbangassina | 2000 | 2005 | Forest | Builtup area | 4.41 |
| Mbangassina | 2000 | 2005 | Builtup area | Builtup area | 9.72 |
| Mbangassina | 2005 | 2010 | Shrubland | Shrubland | 9.54 |
| Mbangassina | 2005 | 2010 | Shrubland | Forest | 2.25 |
| Mbangassina | 2005 | 2010 | Shrubland | Builtup area | 0.45 |
| Mbangassina | 2005 | 2010 | Forest | Shrubland | 0.99 |
| Mbangassina | 2005 | 2010 | Forest | Forest | 1004.76 |
| Mbangassina | 2005 | 2010 | Forest | Builtup area | 4.23 |
| Mbangassina | 2005 | 2010 | Builtup area | Builtup area | 15.12 |
| Mbangassina | 2010 | 2015 | Shrubland | Shrubland | 9.54 |
| Mbangassina | 2010 | 2015 | Shrubland | Forest | 0.45 |
| Mbangassina | 2010 | 2015 | Shrubland | Builtup area | 0.54 |
| Mbangassina | 2010 | 2015 | Forest | Shrubland | 4.05 |
| Mbangassina | 2010 | 2015 | Forest | Forest | 997.38 |
| Mbangassina | 2010 | 2015 | Forest | Builtup area | 5.58 |
| Mbangassina | 2010 | 2015 | Builtup area | Builtup area | 19.8 |
| Mbangassina | 2015 | 2020 | Shrubland | Shrubland | 11.43 |
| Mbangassina | 2015 | 2020 | Shrubland | Forest | 1.98 |
| Mbangassina | 2015 | 2020 | Shrubland | Builtup area | 0.18 |
| Mbangassina | 2015 | 2020 | Forest | Shrubland | 3.87 |
| Mbangassina | 2015 | 2020 | Forest | Forest | 991.17 |
| Mbangassina | 2015 | 2020 | Forest | Builtup area | 2.79 |
| Mbangassina | 2015 | 2020 | Builtup area | Builtup area | 25.92 |
| Mbangassina | 2020 | 2022 | Shrubland | Shrubland | 15.3 |
| Mbangassina | 2020 | 2022 | Forest | Forest | 991.23 |
| Mbangassina | 2020 | 2022 | Forest | Shrubland | 1.92 |
| Mbangassina | 2020 | 2022 | Builtup area | Builtup area | 28.89 |
| Ngam-Kondomeyos | 2000 | 2005 | Shrubland | Forest | 1.44 |
| Ngam-Kondomeyos | 2000 | 2005 | Forest | Forest | 1283.58 |
| Ngam-Kondomeyos | 2005 | 2010 | Forest | Forest | 1285.02 |
| Ngam-Kondomeyos | 2010 | 2015 | Forest | Shrubland | 0.09 |
| Ngam-Kondomeyos | 2010 | 2015 | Forest | Forest | 1284.93 |
| Ngam-Kondomeyos | 2015 | 2020 | Shrubland | Forest | 0.09 |
| Ngam-Kondomeyos | 2015 | 2020 | Forest | Shrubland | 0.18 |
| Ngam-Kondomeyos | 2015 | 2020 | Forest | Forest | 1284.75 |
| Ngam-Kondomeyos | 2020 | 2022 | Shrubland | Shrubland | 0.18 |
| Ngam-Kondomeyos | 2020 | 2022 | Forest | Forest | 1283.9 |
| Ngam-Kondomeyos | 2020 | 2022 | Forest | Shrubland | 0.94 |

**Supplementary Table 4. Socio-demographic contrasts in perceived forest change across nine West African forest patches.** For each country and forest patch, Fisher’s Exact Test (categorical variables) and Kruskal–Wallis H test (ordinal variables) evaluate whether perceptions of forest change differ by gender, marital status, age cohort, community of residence, and education level (sample sizes in parentheses). Degrees of freedom, χ² statistics and exact P-values are reported; blank cells (–) indicate tests that could not be performed because the variable was invariable within the sample. Significance codes: *** P < 0.001; ** P < 0.01; * P < 0.05; · P < 0.1.

| **Country** | **Forest Patch** | **Variable** | **Test** | **Df** | **Chi-squared** | **P-value** | **Significance** | **Significant Difference** |
| --- | --- | --- | --- | --- | --- | --- | --- | --- |
| Togo | Agou (n=351) | Gender | Fisher’s Exact Test | - | - | 0.892054 |  | No |
|  |  | Marital Status | Fisher’s Exact Test | - | - | 0.695652 |  | No |
|  |  | Age | Fisher’s Exact Test | - | - | 0.691154 |  | No |
|  |  | Community | Kruskal-Wallis H test | 7 | 40.835 | 0.0000 | *** | Yes |
|  |  | Education level | Kruskal-Wallis H test | 3 | 0.77808 | 0.8547 |  | No |
|  | Koui (n=150) | Gender | Fisher’s Exact Test | - | - | 0.986007 |  | No |
|  |  | Marital Status | Fisher’s Exact Test | - | - | 0.895552 |  | No |
|  |  | Age | Fisher’s Exact Test | - | - | 0.285857 |  | No |
|  |  | Community | Kruskal-Wallis H test | - | - | - | - | - |
|  |  | Education level | Kruskal-Wallis H test | 3 | 8.6613 | 0.03415 | * | Yes |
|  | Elavagnon-Todji (n=306) | Gender | Fisher’s Exact Test | - | - | 1 |  | No |
|  |  | Marital Status | Fisher’s Exact Test | - | - | 0.708146 |  | No |
|  |  | Age | Fisher’s Exact Test | - | - | 0.866067 |  | No |
|  |  | Community | Kruskal-Wallis H test | 1 | 0.81093 | 0.3678 |  | No |
|  |  | Education level | Kruskal-Wallis H test | 3 | 1.8661 | 0.6007 |  | No |
| Benin | Hlanzoun (n=312) | Gender | Fisher’s Exact Test | - | - | 0.046977 | * | Yes |
|  |  | Marital Status | Fisher’s Exact Test | - | - | 0.974513 |  | No |
|  |  | Age | Fisher’s Exact Test | - | - | 0.067966 | . | No |
|  |  | Community | Kruskal-Wallis H test | 2 | 6.1552 | 0.04607 | * | Yes |
|  |  | Education level | Kruskal-Wallis H test | 3 | 10.639 | 0.01385 | * | Yes |
|  | Ewè-Adakplamè (n=307) | Gender | Fisher’s Exact Test | - | - | 0.031484 | * | Yes |
|  |  | Marital Status | Fisher’s Exact Test | - | - | 0.401799 |  | No |
|  |  | Age | Fisher’s Exact Test | - | - | 0.44078 |  | No |
|  |  | Community | Kruskal-Wallis H test | 2 | 0.48945 | 0.7829 |  | No |
|  |  | Education level | Kruskal-Wallis H test | 3 | 1.025 | 0.7952 |  | No |
| Nigeria | Ikot (n=377) | Gender | Fisher’s Exact Test | - | - | 0.854073 |  | No |
|  |  | Marital Status | Fisher’s Exact Test | - | - | 0.14043 |  | No |
|  |  | Age | Fisher’s Exact Test | - | - | 0.776112 |  | No |
|  |  | Community | Kruskal-Wallis H test | 6 | 16.838 | 0.009899 | ** | Yes |
|  |  | Education level | Kruskal-Wallis H test | 3 | 5.0639 | 0.1672 |  | No |
|  | Iko (n=344) | Gender | Fisher’s Exact Test | - | - | 1 |  | No |
|  |  | Marital Status | Fisher’s Exact Test | - | - | 0.307846 |  | No |
|  |  | Age | Fisher’s Exact Test | - | - | 0.273863 |  | No |
|  |  | Community | Kruskal-Wallis H test | 2 | 5.4097 | 0.06688 |  | Yes |
|  |  | Education level | Kruskal-Wallis H test | 3 | 0.95106 | 0.8131 |  | No |
| Cameroon | Mbangassina (n=302) | Gender | Fisher’s Exact Test | - | - | 1 |  | No |
|  |  | Marital Status | Fisher’s Exact Test | - | - | 0.55922 |  | No |
|  |  | Age | Fisher’s Exact Test | - | - | 0.021989 | * | Yes |
|  |  | Community | Kruskal-Wallis H test | 3 | 3.6093 | 0.3069 |  | No |
|  |  | Education level | Kruskal-Wallis H test | 4 | 4.5562 | 0.3359 |  | No |
|  | Ngam-Kondomeyos (n=172) | Gender | Fisher’s Exact Test | - | - | 0.53923 |  | No |
|  |  | Marital Status | Fisher’s Exact Test | - | - | 0.637681 |  | No |
|  |  | Age | Fisher’s Exact Test | - | - | 0.855073 |  | No |
|  |  | Community | Kruskal-Wallis H test | 1 | 0.00036466 | 0.9848 |  | No |
|  |  | Education level | Kruskal-Wallis H test | 4 | 0.32914 | 0.9879 |  | No |


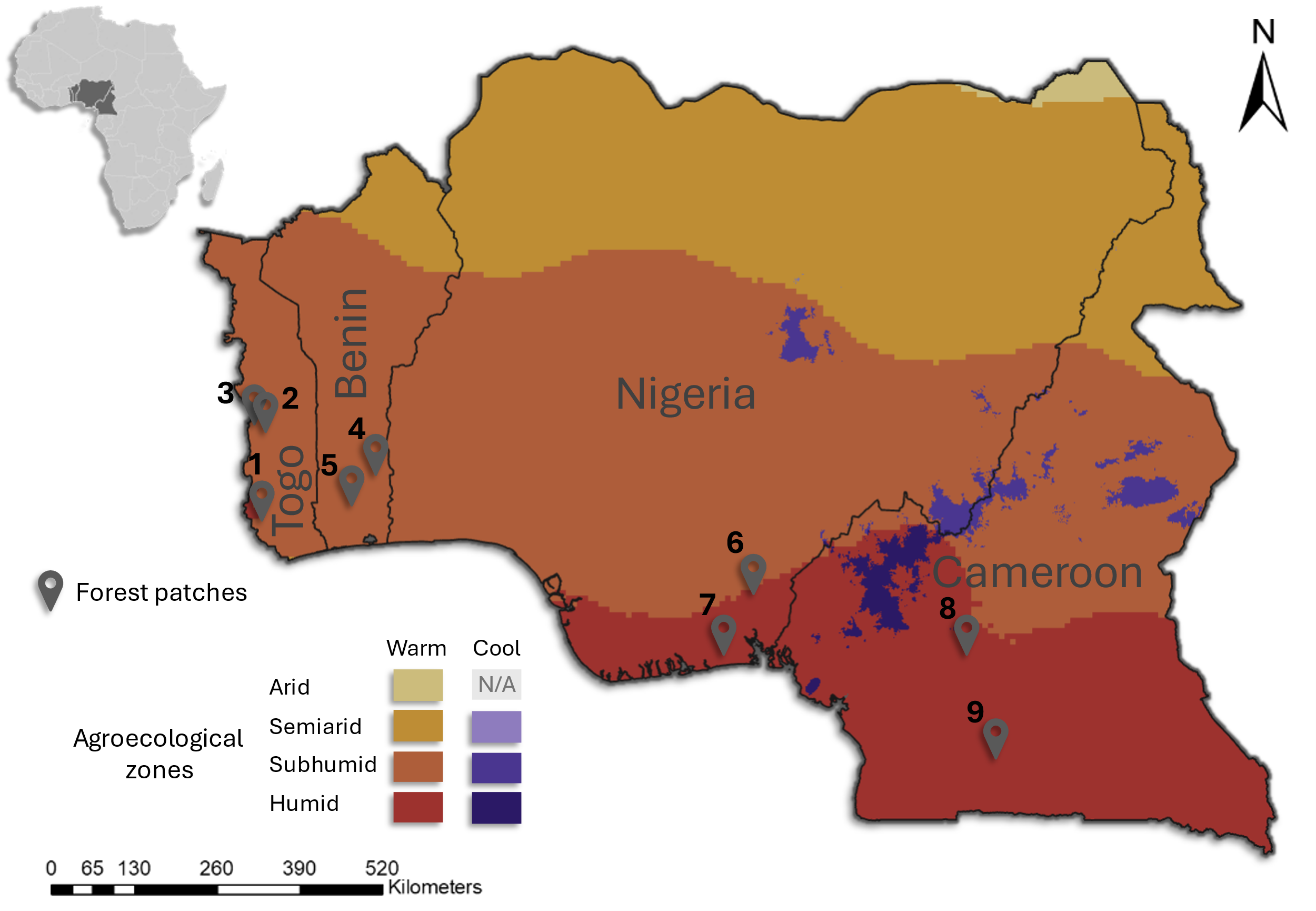


**Supplementary Fig. 1. Geographic setting of the nine focal forest patches in West Africa.** The map locates Agou (1) Elavagnon-Todji (2), and Koui (3) in Togo; Ewè-Adakplamè (4) and Hlanzoun/Lokoli (5) in Benin; Iko (6) and Ikot (7) in Nigeria; and Mbangassina (8) and Ngam-Kondomeyos (9) in Cameroon. Patches fall within humid-warm and sub-humid-warm agro-ecological zones, extracted from HarvestChoice; International Food Policy Research Institute (IFPRI) dataset^1^ (licensed under Creative Commons CC0 1.0 Universal Public Domain Dedication). National boundaries are from the Global Administrative Areas (GADM) database (<https://gadm.org/>). Map created by the authors using ArcGIS Pro v 3.5.2 (<https://www.esri.com/en-us/arcgis/products/arcgis-pro/overview>).

**Supplementary Table 5. Confusion matrix and accuracy statistics for the Landsat-based land-use/land-cover classification (2022; n = 1,500 validation points).** Each cell reports the number of reference samples whose true class (rows) was assigned to a given map class (columns). Producer’s accuracy (with binomial standard error) expresses the proportion of reference samples for a class that were correctly mapped, while user’s accuracy represents the reliability of each mapped class. Overall classification accuracy is 90 % ± 1.31 %.

|  | | Classified | | | | | | | Total | Producer’s Accuracy % (s.e) |
| --- | --- | --- | --- | --- | --- | --- | --- | --- | --- | --- |
|  |  | Shrubland | Built-up | Forest | Sparse Veg. | Wetland | Waterbody | Cropland |  |  |
| Reference Data | Shrubland | 215 | 15 | 10 | 10 | 5 | 0 | 15 | 270 | 79.63 (2.52) |
|  | Built-up | 2 | 195 | 2 | 1 | 2 | 0 | 3 | 205 | 95.12 (1.65) |
|  | Forest | 1 | 1 | 266 | 1 | 0 | 0 | 1 | 270 | 98.52 (1.04) |
|  | Sparse Veg. | 10 | 5 | 5 | 206 | 5 | 0 | 14 | 245 | 84.08 (2.38) |
|  | Wetland | 2 | 2 | 1 | 2 | 181 | 1 | 1 | 190 | 95.26 (1.70) |
|  | Waterbody | 0 | 0 | 0 | 0 | 0 | 154 | 0 | 154 | 100.0 ( 0.00) |
|  | Cropland | 6 | 6 | 5 | 8 | 5 | 3 | 133 | 166 | 80.12 (3.64) |
| Total | | 236 | 224 | 289 | 228 | 198 | 158 | 167 |  | |
| User’s Accuracy % (s.e) | | 91.1 ( 2.58) | 87.05 (2.42) | 92.04 (1.99) | 90.35 (2.50) | 91.41 (2.20) | 97.47 (1.70) | 79.64 (3.63) |  |  |

**Supplementary Table 6. Confusion matrix and accuracy metrics for forest-change classification (2000–2022; n = 1,500 validation points).** Rows correspond to the reference class (forest gain, forest loss, stable forest) and columns to the map class produced by the post-classification change detection. Producer’s accuracy (with binomial standard error, s.e.) measures the proportion of reference pixels in each category that were correctly mapped, whereas user’s accuracy quantifies the reliability of each mapped class. Overall classification accuracy is 92.3 % ± 0.75 %.

|  | | Classified | | | Total | Producer’s Accuracy % (s.e) |
| --- | --- | --- | --- | --- | --- | --- |
|  |  | Forest Gain | Forest Loss | Stable Forest |  |  |
| Reference Data | Forest Gain | 182 | 15 | 23 | 220 | 82.7 (2.55) |
|  | Forest Loss | 12 | 195 | 18 | 225 | 86.7 (2.26) |
|  | Stable Forest | 25 | 22 | 1008 | 1055 | 95.5 (0.64) |
| Total | | 219 | 232 | 1049 |  | |
| User’s Accuracy % (s.e) | | 83.1 (2.53) | 84.1 (2.40) | 96.1 (0.60) |  |  |

# **Reference**

1. HarvestChoice & International Food Policy Research Institute (IFPRI). Agro-Ecological Zones for Africa South of the Sahara. Preprint at https://doi.org/10.7910/DVN/M7XIUB (2015).
